# Intra-subject covariates of QT variability indices: influence of ECG noise

**DOI:** 10.3389/fphys.2026.1926685

**Published:** 2026-09-21

**Authors:** Jan Řehoř, Kateřina Helánová, Martina Šišáková, Tomáš Novotný, Irena Andršová, Marek Malik

**Affiliations:** 1 Department of Internal Medicine and Cardiology, University Hospital Brno, Brno, Czechia; 2 Department of Internal Medicine and Cardiology, Faculty of Medicine, Masaryk University, Brno, Czechia; 3 National Heart and Lung Institute, Imperial College, London, United Kingdom

**Keywords:** electrocardiographic noise, healthy subjects, heart rate variability, individual multivariable regressions, postural testing, QT variability

## Abstract

**Background/objectives:**

Electrocardiogram (ECG) assessment of increased beat-to-beat QT interval variability indices has repeatedly been proposed to indicate adverse outcomes in different clinical populations. Nevertheless, the reported values of the indices appear poorly reproducible. Consequently, there is no clear consensus on the physiologic normal values. The limited stability of the indices might be linked to their relation to heart rate and other ECG factors such as the underlying heart rate variability. We have therefore investigated covariates of QT variability indices to answer the question of whether correction for covariates might, in principle, increase the reproducibility.

**Methods:**

In a population of 251 healthy subjects (mean age 33.6 ± 9.1 years, 108 females) who underwent repeated provocative manoeuvres, ECG measurements were performed in recordings obtained during different postural positions. In each subject, measurements of QT variability and of its plausible covariates were made in 32 separate sections of the recordings. The measurements included mean normal-to-normal RR intervals, mean QT intervals, standard deviations of RR and QT intervals, and root mean square of successive differences of QT and RR intervals. In each repeated postural position, the measurements were made in 5-minute intervals of stable heart rate. Electrocardiographic noise was assessed based on signal-to-noise ratios. Univariable and multivariable regression analyses were performed in each subject separately to assess the stability of QT variability relationship to the covariates.

**Results:**

Univariably, QT variability indices were significantly more strongly related to ECG noise than to other covariates. The regression coefficients relating QT variability to other covariates were also highly variable. In multivariable regression analyses with backwards elimination, ECG noise remained a statistically significant covariate of QT variability indices in approximately one third and one half of the population in the regression analyses of standard deviation of QT intervals and of root mean square of successive QT differences, respectively.

**Conclusions:**

It is not feasible to reduce the inconsistency of QT variability expressions by correcting them for other ECG measurements. When QT variability measures are used in clinical studies, e.g., studies of risk prediction, influence of ECG noise needs to be considered. Multivariable regression models of QT variability need to involve ECG noise data to eliminate unfounded conclusions.

## Introduction

The 12-lead clinical electrocardiogram (ECG) is commonly obtained in almost all patients irrespective of their medical conditions and of the facility involved in their medical review and/or treatment. In addition, ECG is also a routine part of regular health assessment programmes. This makes the ECG signals suitable for wide-range screening that involves not only standard visual diagnostics (such as the visible ischaemic changes, rhythm abnormalities, and channelopathies) but also detection of abnormalities beyond regular ECG assessment (Malik, 2024).

Novel risk-assessment methods have recently been proposed based on artificial intelligence (Chang et al., 2025; Croon et al., 2025; Swain et al., 2025). Nevertheless, these still complement those based on ECG morphology (Hnatkova et al., 2022; Hnatkova et al., 2024) and on beat-to-beat changes (Tachmatzidis et al., 2021; Ebrahimian et al., 2024; Rahola et al., 2024). Many of the risk studies investigating beat-to-beat changes involve examinations of heart rate variability (HRV) (Task Force of the European Society of Cardiology and the North American Society of Pacing and Electrophysiology, 1996). Very large numbers of HRV observations have been reported involving almost all imaginable physiologic situations and clinical conditions (Steger et al., 2021; Jarczok et al., 2022; Rooney et al., 2024). Nonetheless, other beat-to-beat changes have also been investigated. In this text, we describe a study that examined specific aspects of beat-to-beat changes in the QT interval duration.

Clinical implications of QT interval variability were introduced in the 1997 seminal publication (Berger et al., 1997) that proposed a composite formula relating the beat-to-beat variance of QT interval duration not only to the mean QT duration but also to the variance of underlying RR intervals again normalised for the mean RR duration. This so-called QT variability index was subsequently adopted by many investigators and linked not only to cardiac risk (Carney et al., 2003; Chen et al., 2021; Sato et al., 2025) but also to many different conditions (Ernst et al., 2023; Ebrahimian et al., 2024; Bergdahl et al., 2026). Recent cellular computation models also suggested that QT interval variability might detect cellular mechanisms responsible for arrhythmias (Sato et al., 2025).

The assessment, physiological interpretation, and clinical implication of QT interval variability have been the topics of a standardisation document (Baumert et al., 2016) that, among others, reviewed a variety of indices that have previously been used to express the QT variability. In addition to the QT variability index, simple statistical expressions of QT duration changes have been used in different studies.

Still, the relationship between QT interval variability indices and the associated HRV expressions appears different between different healthy subjects (Řehoř et al., 2026a). This is likely, at least in part, related to the influence of ECG noise on QT variability assessment (Rijnbeek et al., 2017; Orini et al., 2018; Toman et al., 2022). This problem includes considerations of the relationship between heart rate and ECG noise which is known to be higher in recordings obtained under heart rate increasing conditions.

The between-subject differences in the dependence of QT variability on the underlying heart rate and on standard time-domain HRV expressions lead logically to the question of whether heart rate and/or HRV corrections of QT variability should be considered similar to the correction of QT interval duration for the underlying heart rate (Valentinuzzi, 1971; Hnatkova and Malik, 1999). While such a concept has been introduced in the initial definition of the QT variability index (Berger et al., 1997), the way in which the QT variability index incorporates the correction has not been scrutinised. Optimisation of any correction formula requires deeper insight. It seems also plausible to speculate that correcting QT variability for heart rate and/or HRV in an optimal way might reduce the dependency on ECG noise.

The experience with heart rate correction of the QT interval also shows that the dynamic properties of ECG intervals (Malik et al., 2013; Andršová et al., 2021) and other ECG-derived factors (Ono et al., 2006; Hnatkova et al., 2019b; Toman et al., 2020) are highly individual. Therefore, to avoid the possibility of substantially biased results, it would not be appropriate to test the heart rate and other possible corrections of QT variability on a population basis (Malik et al., 2019).

To address these problems, we are now reporting a study that investigated the relationship of elementary measures of QT variability to plausible ECG-derived covariates including ECG noise. We have analysed data with repeated measurements in different healthy subjects that allowed us to address the QT variability covariates on an intra-individual basis.

## Methods

### Study population

As previously reported (Hnatkova et al., 2019a; Andršová et al., 2022), a large clinical pharmacology study enrolled 352 healthy subjects (176 females). The enrolment procedures satisfied standard criteria for phase I clinical investigations. The screening phase of the study confirmed normal clinical ECGs and normal standard clinical investigations (ICH Guideline, 2001) in all participants. Importantly, this excluded subjects with cardiac and/or neurological abnormalities. During the study, no consumption of alcohol, and/or caffeinated drinks was permitted. The subjects were also not allowed to smoke. Female subjects had a negative pregnancy test and were not on hormonal contraceptives. The clinical pharmacology study was conducted at two centres and was approved by corresponding ethics boards (Parexel, Baltimore, MD, USA; and California Clinical Trials, Glendale, CA, USA). Written informed consent was signed by each participant. The consent included the option of future research data explorations. The study protocol and the informed consent satisfied the prerequisites of the Helsinki declaration. Only anonymised off-treatment data were used in the present investigation. Therefore, further details of the clinical pharmacology study are of no consequence.

### Postural provocations

The clinical pharmacology study included 2 periods separated by sufficient wash-out intervals. Each of these periods included 2 drug-free days. During each of these drug-free days, the study protocol prescribed 2 separate sequences of postural provocations. Each of these was composed of 4 positions. Whilst the first sequence was composed of (in this order) supine → sitting → standing → supine, the second sequence changed the order of positions to supine → standing → sitting → supine. All the positions were maintained without any external visual, acoustic and/or mechanical interference and without any body movement apart from respiration related chest changes. All positions were maintained for 10 minutes except for the standing positions that were maintained for 15 minutes. The position changes were prompted by verbal instructions and were, per protocol, completed within 20 seconds.

### Electrocardiography recordings and measurements

During investigative days of the clinical pharmacology study (that is including the drug-free days) continuous 12-lead Holter recordings were obtained. The present investigation concerned the portions of these Holter recordings that covered the postural provocation episodes. Following previously reported methods (Malik et al., 2008; Malik et al., 2012), separate QRS complexes were recognised, and their morphology classified to differentiate those of sinus nodal origin from others. RR intervals were measured at a 1 kHz resolution. Cross-correlation maxima over individual leads provided systematic timing of QRS complexes.

The continuous recordings were divided into a sequence of 10-second segments with neighbouring segments overlapping by 5 seconds. In each segment, representative beat-form was constructed for each ECG lead. Standard median method was applied to individual QRS-T patterns (Cuesta-Frau et al., 2003). Within each postural position of each repeated postural provocative test, at least 5 non-overlapping and non-adjacent 10-second ECG segments were selected. Previously tested methods (Malik et al., 2008; Malik et al., 2012) identified QRS onset and T wave offset in the representative median beat-forms of these ECG segments. The segment-to-segment consistency of the delineation positions was assured (Hnatkova et al., 2009; Johannesen et al., 2014). Cross-correlation method for pattern identification (Malik et al., 1996) consistent with the original proposal for QT variability assessment (Berger et al., 1997) projected the delineation measurements to representative beatforms of all ECG segments recorded during the postural provocative test. The same technique (Malik et al., 1996; Berger et al., 1997) was subsequently employed to obtain the QRS onset and T wave offset in all cardiac cycles. Repeated visual control of a number of sample segments subsequently validated the individual ECG beat measurements. The 1 kHz sample resolution provided the measurements with 1 millisecond (ms) precision.

### Electrocardiographic data

Only subjects with complete data were included in the present analysis. That is, all postural provocation data were excluded if the subject did not complete any of the tests because of study drop-out, pre-syncopal symptoms, substantial discomfort, or other reasons. Data were further excluded if the QT interval measurements were compromised because of excessive noise (e.g. due to temporarily disconnected ECG leads). In each subject and in each postural position, 5-minute sub-interval was selected with minimal heart rate spread (i.e., minimal difference between heart rates measured in 30-second intervals). If it became impossible to find such 5-minute sub-intervals free of ventricular and/or supraventricular ectopic beats for all postural positions in a given subject, the data of the subject were excluded.

Thus, in each subject eligible for the present analysis, we identified ectopic-free 5-minute sub-intervals in all 8 postural episodes of each of the 4 drug-free baseline days. In every such sub-interval, we calculated the mean normal-to-normal RR interval (NN interval), mean QT interval duration, standard deviation of all NN intervals (SDNN), root mean square of successive differences between neighbouring NN intervals (RMSSD-NN), standard deviation of all QT intervals (SDQT), and root mean square of successive differences between neighbouring QT intervals (RMSSD-QT). That is, the RMSSD-xx parameters were calculated according to the formula where the sequence ${\left\{xx_{i}\right\}}_{i=0}^{n}$ represents either the consecutive NN intervals or the consecutive QT intervals measured in the ectopic-free 5-minute sub-interval of a postural position. All the measurements were expressed in ms.


$$RMSSDxx=\;\sqrt[{2}]{\frac{1}{n}\sum_{i=1}^{n}{\left(xx_{i}-xx_{i-1}\;\right)}^{2}}$$

### Electrocardiographic noise

To investigate the influence of recording noise on the measurement precision, signal-to-noise ratio was determined using the method previously proposed by Galeotti et al (Johannesen and Galeotti, 2012; Galeotti et al., 2013). Specifically, in each of the selected ectopic-free 5-minute sub-intervals, this approach divided the ECG noise into three components, namely (a) the baseline wander (i.e., changes in the baseline signal, mainly due to respiration and/or electrode movement artefacts), (b) the power-line interference (caused by the noise from the power distribution network) and (c) residual noise (arising from the myoelectric potentials of skeletal muscles). The baseline wander was determined using the ideally isoelectric points within the PQ segment of each beat. The power-line interference was estimated from the known frequency of power lines. The residual noise was finally determined by subtracting the ideal representative waveforms from the signal after the baseline wander and power interference were eliminated, i.e. by a method originally proposed by Batchvarov et al. (2002).

The sample-by-sample root mean square difference between the original and the filtered signal was related to the root mean square value of the filtered signal. This provided the value of signal-to-noise ratio. The reciprocal value of the ratio was used as the estimate of ECG signal noise (unitless value expressed in per-cent).

### Within-subject regression analyses

As described, the postural changes [supine → sitting → standing → supine] and [supine → standing → sitting → supine] were repeated on 4 different drug-free study days. When values of mean NN, mean QT, SDNN, SDQT, RMSSD-RR, RMSSD-QT, and ECG noise were obtained for each postural position, 32 separate readings of these ECG indices were available for each study subject with complete data (as defined in the section on electrocardiographic data).

We were thus able to investigate uni- and multivariable regression analyses between the QT variability indices and their covariates in each subject separately. That is, for each subject S, the corresponding values of QT variability (or other ECG-derived) indicator {QkS}k=132, and the corresponding values of a set of values of *n* covariates {{CkSi}i=1n}k=132, where *n* ≪ 32, we investigated the relationship where ${\left\{{}^{S}\beta_{i}\right\}}_{i=0}^{n}$ are subject-specific regression coefficients, and ϵkS are instances of zero-centred subject-specific regression variance. The standard linear regression model also provided statistical significances ${\left\{{}^{S}{𝒫}_{i}\right\}}_{i=0}^{n}$ of coefficients ${\left\{{}^{S}\beta_{i}\right\}}_{i=0}^{n}$.


{QkS= Sβ0+∑i=1nSβiCkSi+ϵkS}k=132

When repeating these investigations for all study subjects, the inter-subject stability of regression coefficients ${\left\{{}^{\cdots }\beta_{i}\right\}}_{i=0}^{n}$ and of significances ${\left\{{}^{\cdots }{𝒫}_{i}\right\}}_{i=0}^{n}$ answered the question of whether the characteristic Q  might reasonably be corrected for the combination of covariates ${\left\{C_{i}\right\}}_{i=1}^{n}$.

### Data presentation and statistics

Basic continuous data with expected normal distribution are presented as mean ± standard deviation (SD); categorical data are presented as counts and percentages. Intra- and inter-subject comparisons were based on Wilcoxon signed rank test, and Kolmogorov-Smirnov test, respectively. Where appropriate non-parametric Spearman correlation coefficients were used. P values above 0.05 were considered statistically non-significant (NS).

Regression analysis was performed in five steps designed to understand the influence of ECG noise when used univariably and when used together with other covariates in gradually extended multivariable settings. The five steps were as follows: (1) To understand the background of subsequent analyses, SDNN and RMSSD-NN were univariably related to mean NN and to ECG noise. (2) Univariable QT variability analyses involved relations of SDQT and RMSSD-QT to mean NN, SDNN, RMSSD-NN, mean QT, and ECG noise. (3) Bivariable QT variability analyses related SDQT and RMSSD-QT to a combination of ECG noise with each of mean NN, SDNN, RMSSD-NN, and mean QT. (4) Multivariable analyses related SDQT to four covariate triplets, namely mean NN & SDNN & ECG noise, mean NN & RMSSD-NN & ECG noise, mean NN & mean QT & ECG noise, and SDNN & mean QT & ECG noise. Similar analyses of this step related RMSSD-QT to four covariate triplets of mean NN & SDNN & ECG noise, mean NN & RMSSD-NN & ECG noise, mean NN & mean QT & ECG noise, and RMSSD-NN & mean QT & ECG noise. (5) Finally, the full multivariable analyses related SDQT to a covariate quadruplet of mean NN & SDNN & mean QT & ECG noise, and similarly RMSSD-QT to a quadruplet of mean NN & RMSSD-NN & mean QT & ECG noise.

The intra-subject multivariable regression analyses were performed with stepwise backwards elimination. For selected covariates, the regression coefficients ${\left\{{}^{\cdots }\beta_{i}\right\}}_{i=0}^{n}$ and the corresponding p-values ${\left\{{}^{\cdots }{𝒫}_{i}\right\}}_{i=0}^{n}$ were displayed graphically both in scatter diagrams and in cumulative distributions.

ECG processing and calculations of the ECG indices were made using internally validated packages in C++ (Microsoft Visual Studio 2025, 64-bit version 17.14.14) and in Python (64-bit, version 3.12.4), statistical comparisons were made using SPSS package (IBM, version 31.0.0.0). Because of the interdependence between analysed indices, no adjustment for multiplicity of statistical testing was employed.

## Results

### Population and electrocardiographic data

After excluding subjects who did not complete the source study and those who did not have suitable ECG recordings, this investigation analysed ECG data of 251 subjects. Of these, 108 were females aged 34.2 ± 10.2 years, and 143 were males aged 33.2 ± 8.2 years (no significant difference between the ages of the sex groups).

Sets of 32 separate values of mean NN, mean QT, SDNN, SDQT, RMSSD-NN, RMSSD-QT, and ECG noise were available for each analysed subject.

The ECG noise levels differed in different postural positions and between females and males. Their values were (females vs males): 5.51 ± 0.67% vs 5.14 ± 0.58% (p<0.0001), 6.20 ± 1.03% vs 5.78 ± 1.01% (p=0.001), and 6.90 ± 2.00% vs 6.07 ± 1.11% (p=0.0001), in supine, sitting, and standing positions, respectively. Other measured ECG parameters and their sex differences are shown in **Supplementary Table 1**.

### Regression analysis of HRV covariates

**Figure 1** shows the results of univariable regression analyses relating SDNN and RMSSD-NN to mean NN. (That is, in each subject, SDNN was related to mean NN, and separately, RMSSD-NN was related to mean NN.)

**Figure 1:**
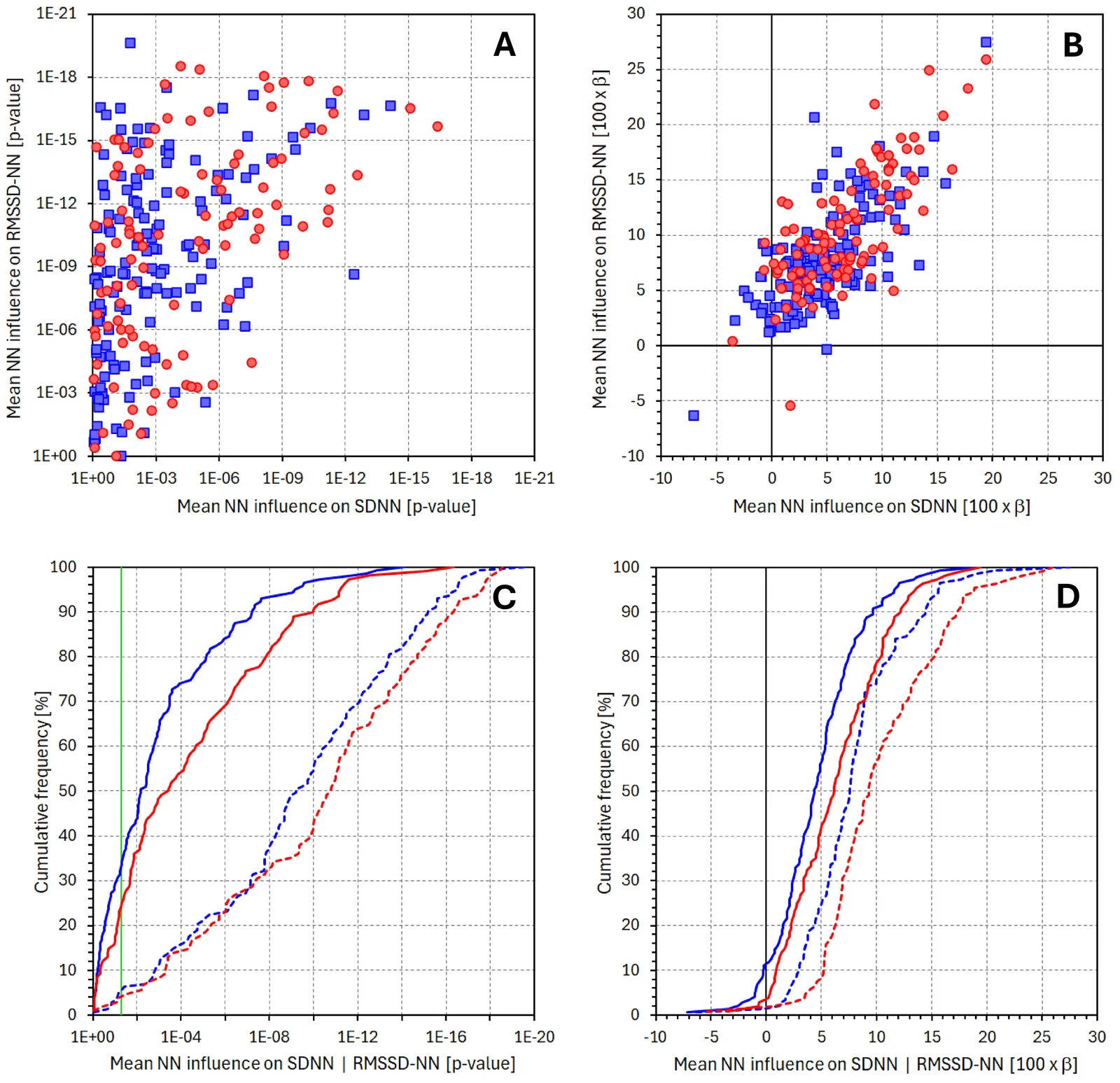
Results of regression analyses relating SDNN and RMSSD-NN values to mean NN intervals. For both univariable regressions, Panels **(A, B)** show scatter diagrams of p-values of *β* coefficients, and of *β* coefficients, respectively. Red circles and blue squares correspond to individual female and male subjects, respectively. Panels **(C, D)** show cumulative frequences of data shown in the scatter diagrams [p-values of *β* coefficients in Panel **(C)** and *β* coefficients in Panel **(D)**]. Bold lines correspond to the SDNN/mean NN regressions, dashed lines to the RMSSD-NN/mean NN regressions; red and blue lines correspond to the distributions in female and male subjects, respectively. Panel **(C)** also shows the threshold of statistical significance (vertical green line). Note that the graphical interpretation of Panel **(C)** is potentially counter-intuitive: the lower the curve, the more frequent strongly significant results.

Panel A of the Figure shows scatter diagram between statistical significances SDNN vs mean NN and RMSSD-NN vs mean NN. Visually, it is obvious that the statistical significance RMSSD-NN vs mean NN tended to be stronger than the significance of SDNN vs mean NN. This is confirmed in Panel C of the Figure (p<0.0001 in both females and males). Note also in Panel C that RMSSD-NN was not significantly dependent on mean NN in 4 females (3.7%) and 7 males (4.9%). For SDNN, these numbers were 26 (24.1%) and 47 (32.9%), respectively. Panel B of the Figure shows the scatter diagram of *β* coefficients. Their cumulative distribution is shown in Panel D. *β* coefficients of the RMSSD-NN/mean NN regression tended to be higher than those of SDNN/mean NN regression (p<0.0001 for both sexes). Also, the *β* coefficients were higher in females compared to males (p<0.0001 for both RMSSD-NN/mean NN and SDNN/mean NN).

**Figure 2** shows the results of corresponding analyses relating SDNN and RMSSD-NN to ECG noise. As seen in Panels A and C, statistical significance of noise influence on RMSSD-NN was stronger than that of SDNN (p<0.0001 in females, p< 0.005 in males). Nevertheless, the significance was rather weak. The influence of ECG noise on RMSSD-NN was not statistically significant in 58 females (53.7%) and in 79 males (55.2%). For the influence of ECG noise on SDNN, these numbers were 82 females (75.9%) and 98 males (68.5%). Panels B and D show that the influence of ECG noise on both RMSSD-NN and, to some lesser extent, on SDNN was mainly negative in the sense that increased noise levels were associated with lower RMSSD-NN and SDNN values. The medians of *β* values were significantly negative in females for both SDNN (p=0.011) and RMSSD-NN (p<0.0001), and in males for RMSSD-NN (p<0.0001). That is, in the majority of subjects, as the ECG noise increased, SDNN and RMSSD-NN decreased. This corresponds to the known increase of ECG noise during postural positions that are also associated with increased heart rate (Hnatkova et al., 2019a; Hamada et al., 2022).

**Figure 2:**
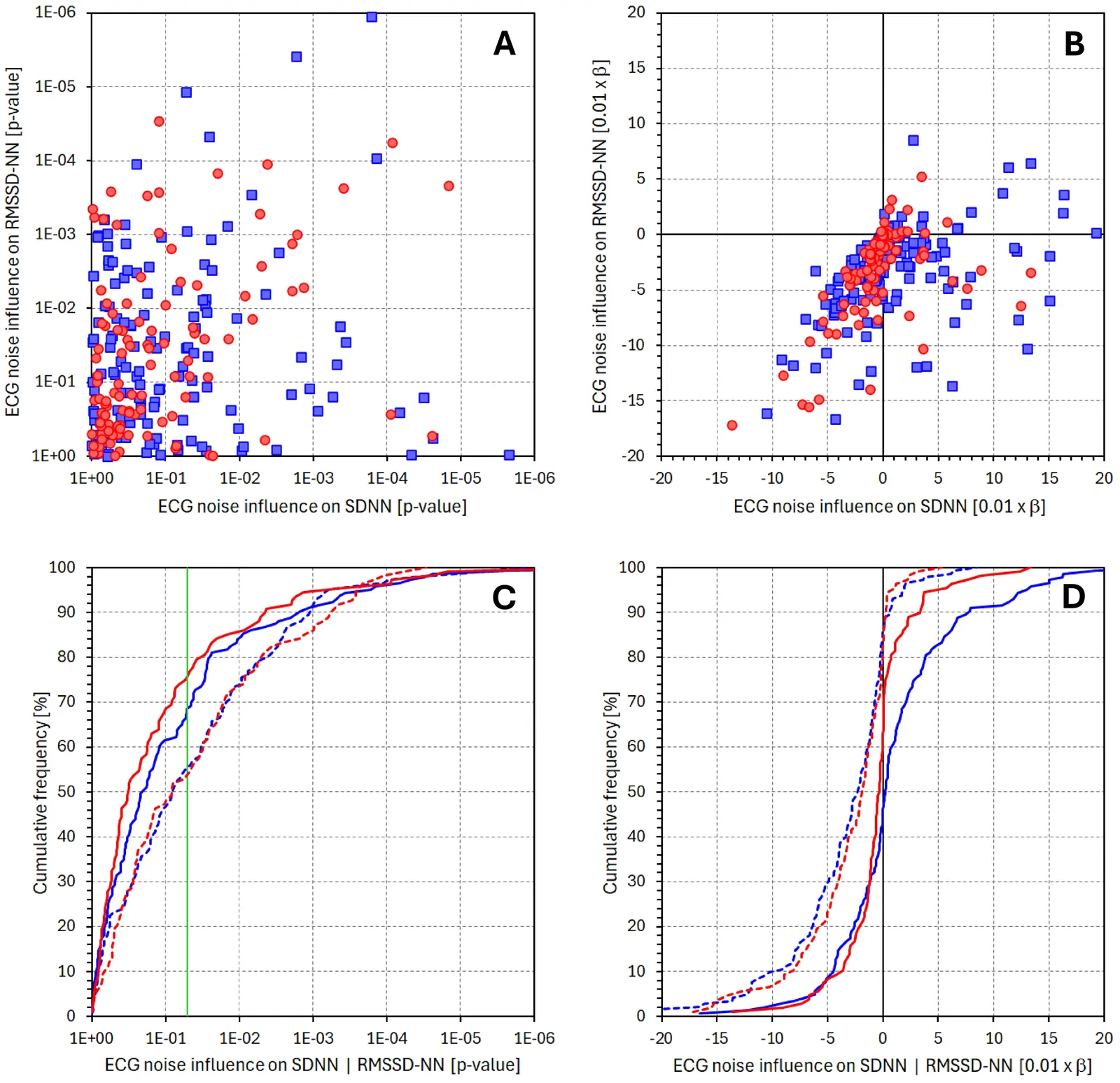
Results of regression analyses relating SDNN and RMSSD-NN values to ECG noise. The layout and meaning of individual panels correspond to the layout and meaning of **Figure 1**.

### Univariable regressions of QT variability indices

**Figure 3** shows the scatter diagrams of statistical significances of univariable regression analyses of SDQT and RMSSD-QT. Visual comparison shows that SDNN/RMSSD-NN (Panel B) and mean QT interval (Panel C) were much weaker covariates compared to mean NN (Panel A) and particularly to ECG noise (Panel D). This is confirmed in **Figure 4** that shows the cumulative distributions of these statistical significances in corresponding panels A to D. SDNN as covariate of SDQT was nonsignificant in 78 females (72.2%) and 81 males (56.4%). Mean QT interval was a nonsignificant covariate of SDQT in 54 females (50.0%) and 81 males (56.6%). For RMSSD-QT, the corresponding numbers were 50 females (46.3%) and 85 males (59.4%). The mean NN interval was only slightly stronger covariate, nonsignificant contributor to SDQT in 53 females (49.1%) and 79 males (55.2%). For RMSSD-QT, the corresponding numbers were 49 females (45.4%) and 82 males (57.3%). The ECG noise was, in these univariable regressions, the strongest covariate of both SDQT and RMSSD-QT, with nonsignificant counts of 47 females (43.5%) and 55 males (38.4%) for SDQT, and 42 females (38.9%) and 55 males (38.4%) for RMSSD-QT.

**Figure 3:**
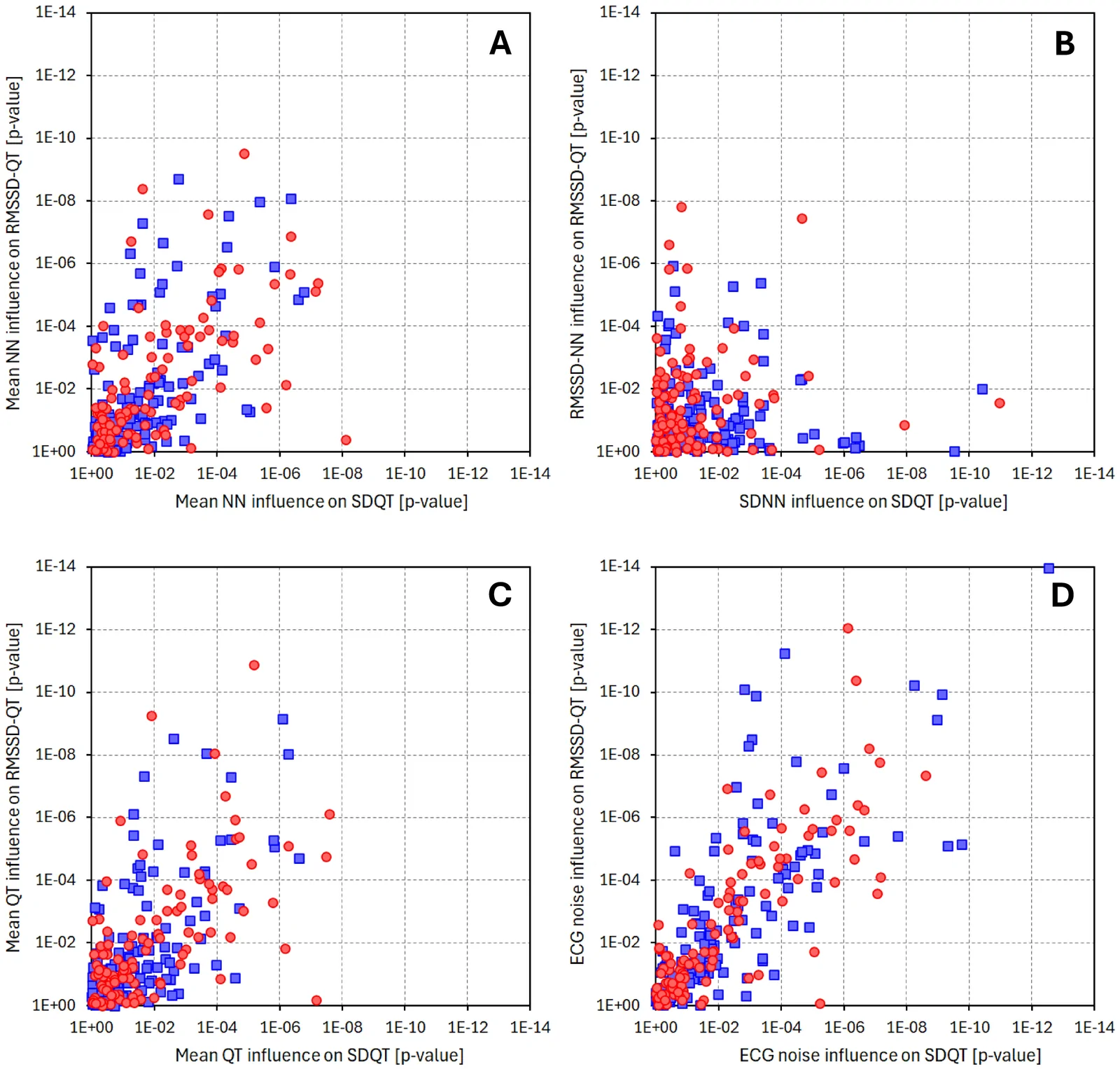
Results of the univariable regression analyses relating SDQT and RMSSD-QT values to mean NN intervals **(A)**, SDNN and RMSSD-NN **(B)**, mean QT intervals **(C)** and ECG noise **(D)**. The layout of all panels and meaning of symbols correspond to the layout and symbols of Panel **(A)** of **Figure 1**.

**Figure 4:**
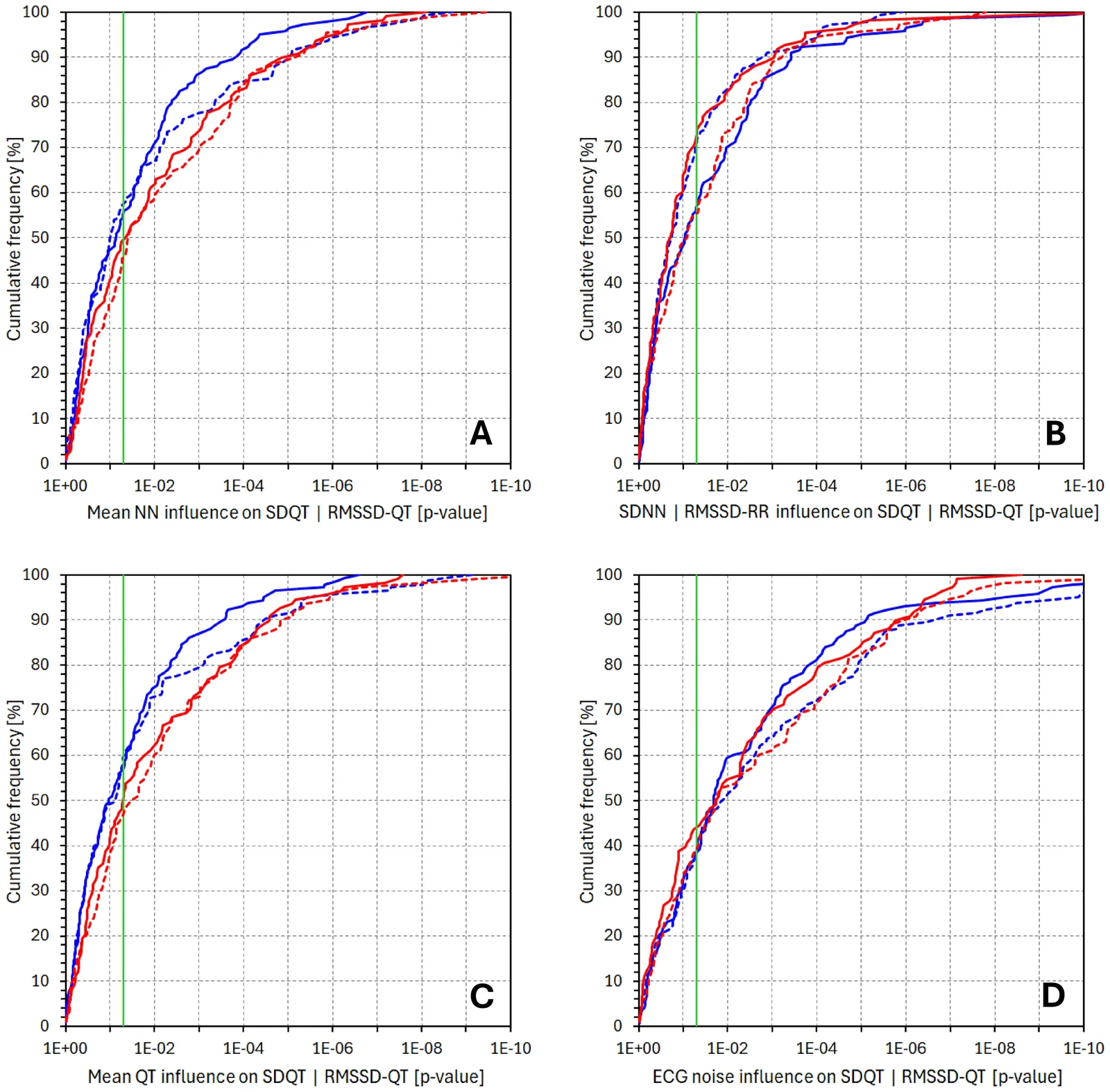
Results of the univariable regression analyses relating SDQT and RMSSD-QT values to mean NN intervals **(A)**, SDNN and RMSSD-NN **(B)**, mean QT intervals **(C)** and ECG noise **(D)**. The layout of all panels and meaning of symbols correspond to the layout and symbols of Panel C of **Figure 1**. Bold and dashed lines correspond to the regression results of SDQT and to the regression results of RMSSD-QT, respectively.

The scatter diagrams of *β* coefficients of these univariable regression analyses are shown in **Figure 5** (with same layout of panels). We note that for both SDQT and RMSSD-QT, the mean NN *β* coefficients were mostly negative, similar to the mean QT *β* coefficients. The *β* coefficients of SDQT/SDNN and RMSSD-QT/RMSSD-NN were somewhat erratic, and the coefficients of ECG noise were mostly positive. This observation corresponds to the cumulative distributions of the *β* coefficients shown in corresponding panels of **Figure 6**. All the *β* coefficients except the SDQT/SDNN regression were significantly negative (p< 0.0001 for both sexes). On the contrary, SDQT/SDNN *β* coefficients were statistically positive (p = 0.002 in females and p< 0.0001 in males). Significant sex differences in the *β* coefficients were found only in the regressions relating QT variability indices to mean NN and to mean QT (all p< 0.001 except p = 0.004 for the RMSSD-QT/mean QT regressions).

**Figure 5:**
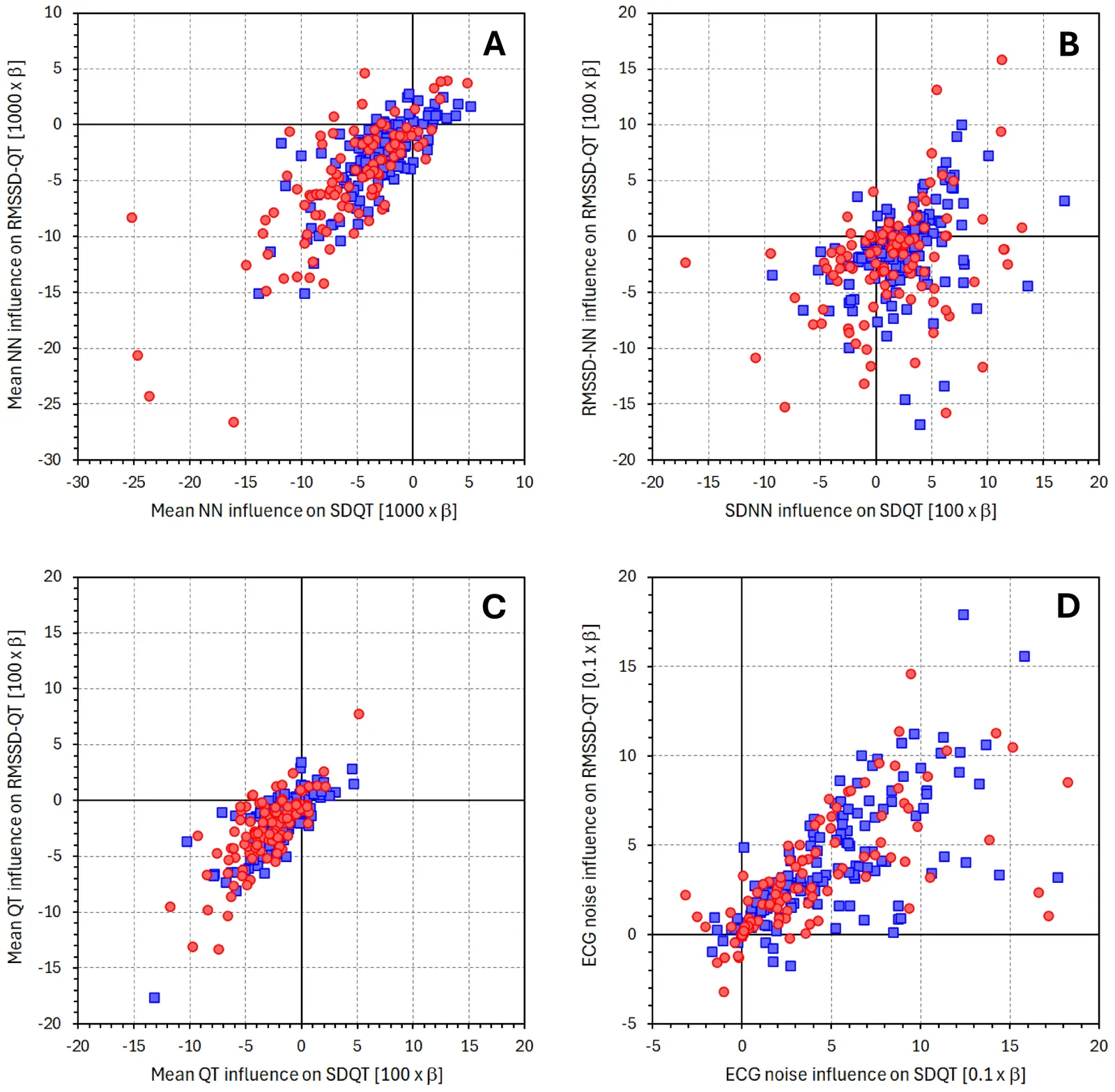
Results of the univariable regression analyses relating SDQT and RMSSD-QT values to mean NN intervals **(A)**, SDNN and RMSSD-NN **(B)**, mean QT intervals **(C)** and ECG noise **(D)**. The layout of all panels and meaning of symbols correspond to the layout and symbols of Panel B of **Figure 1**.

**Figure 6:**
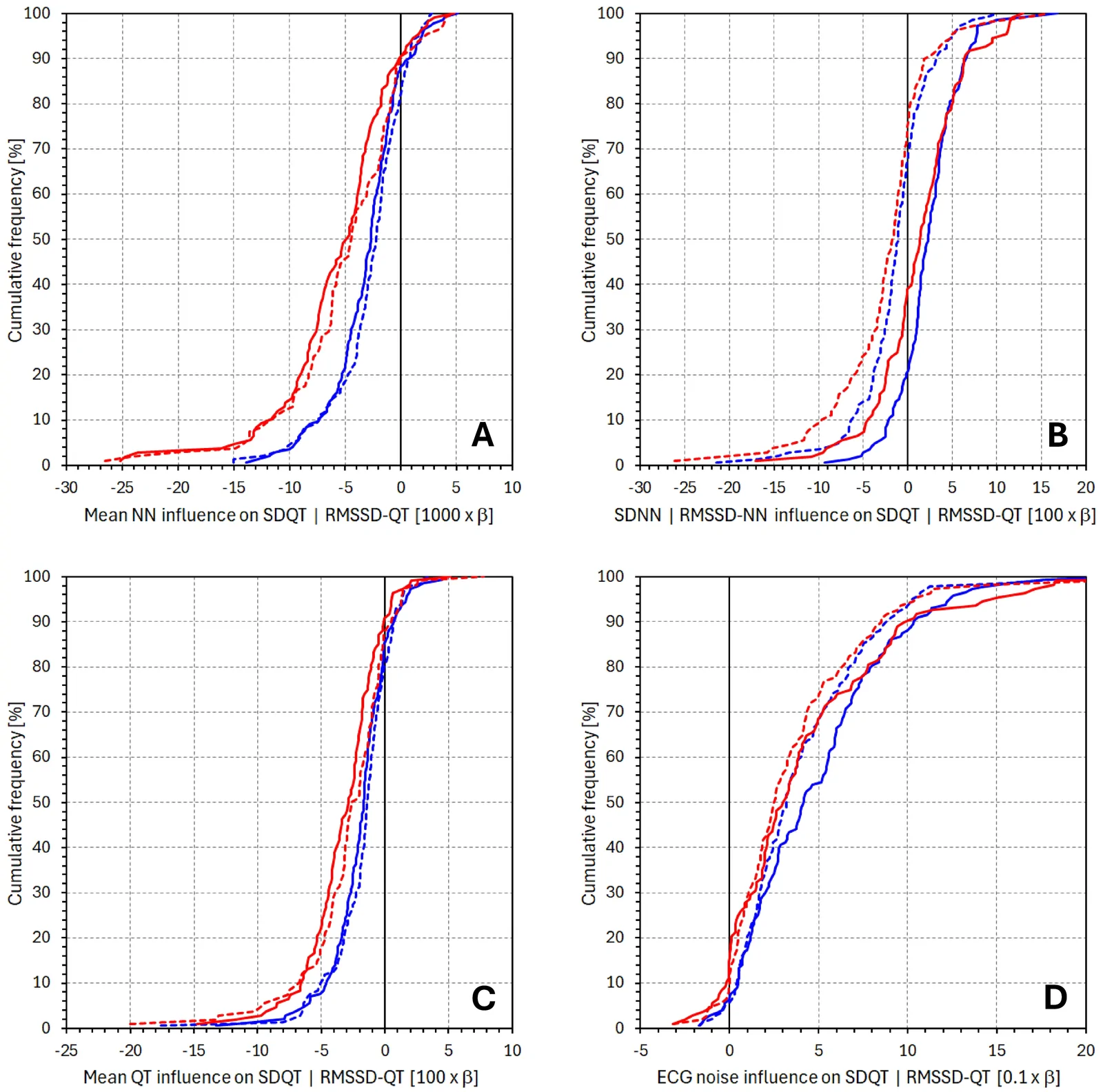
Results of the univariable regression analyses relating SDQT and RMSSD-QT values to mean NN intervals **(A)**, SDNN and RMSSD-NN **(B)**, mean QT intervals **(C)** and ECG noise **(D)**. The layout of all panels and meaning of symbols correspond to the layout and symbols of Panel D of **Figure 1**. Bold and dashed lines correspond to the regression results of SDQT and to the regression results of RMSSD-QT, respectively.

### Bivariable regressions of SDQT

The distribution of statistical significances of the bivariate SDQT regressions is shown in **Figure 7**, their cumulative distributions are shown in **Figure 8**. While **Figure 8** suggests that in all tested bivariate models, ECG noise outperformed other SDQT covariates, paired comparisons are more important. These showed that in males, the median of noise-related p-values was always lower (i.e., showing stronger association) than the median of other covariates (p< 0.001 for all cases). In females, this was only the case in SDQT regressions to SDNN and to RMSSD-NN (p< 0.001 in both cases) while for SDQT regressions to mean NN and to mean QT, the difference was not significant.

**Figure 7:**
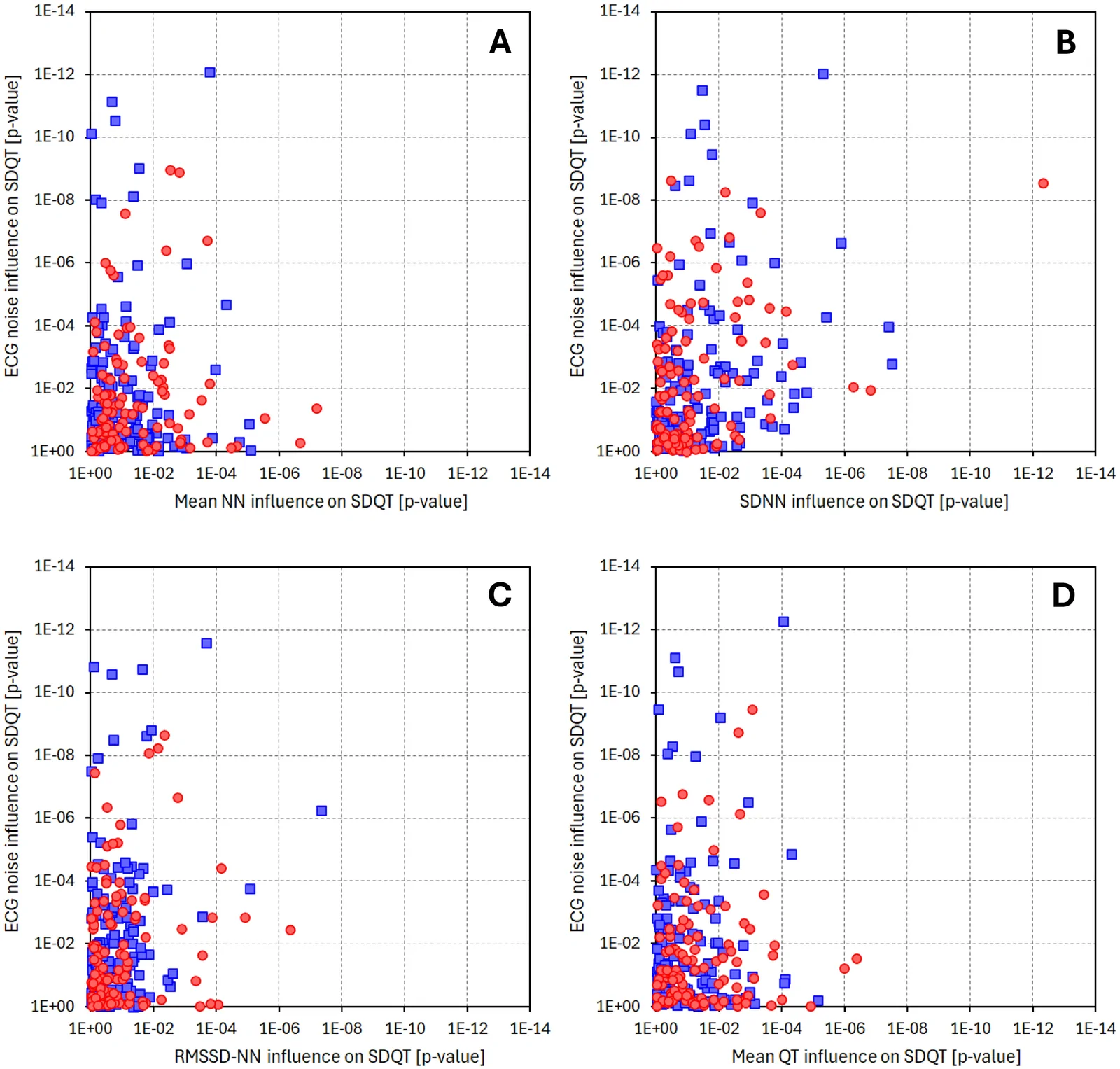
Results of the bivariable regression analyses relating SDQT values to the combination of ECG noise with mean NN intervals **(A)**, SDNN **(B)**, RMSSD-NN **(C)**, and mean QT intervals **(D)**. The layout of all panels and meaning of symbols correspond to the layout and symbols of Panel A of **Figure 1**. In all panels, the regression contributions of ECG noise are shown on the vertical axis, the contributions of the other covariate on the horizontal axis.

**Figure 8:**
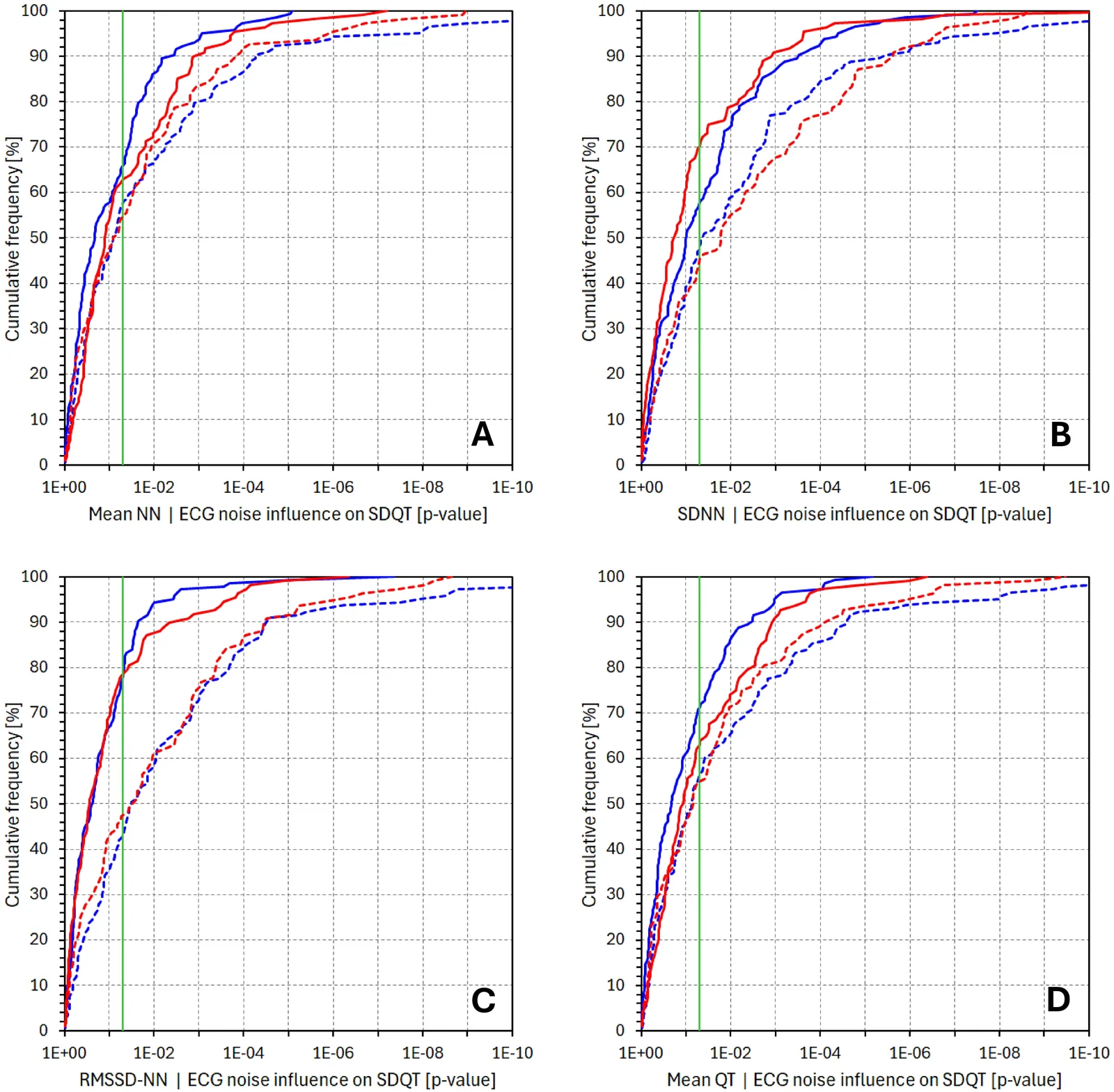
Results of the bivariable regression analyses relating SDQT values to the combination of ECG noise with mean NN intervals **(A)**, SDNN **(B)**, RMSSD-NN **(C)**, and mean QT intervals **(D)**. The layout of all panels and meaning of symbols correspond to the layout and symbols of Panel C of **Figure 1**. In all panels, the dashed lines correspond to the contributions of ECG noise, the full lines to the contributions of the other covariate.

**Figure 9** shows the scatter diagrams of *β* coefficients corresponding to the ECG noise versus the *β* coefficients of other SDQT covariates (that is, the *β* coefficients of the initial bivariable step of the analyses). In individual bivariate analyses, the *β* coefficients of ECG noise were weakly correlated to the coefficients of other covariates. In females, the correlations of the SDQT regressions to ECG noise combined with mean NN, SDNN, RMSSD-NN, and mean QT were 0.353 (p< 0.001), -0.004 (NS), 0.194 (p = 0.044), and 0.304 (p = 0.001), respectively. The corresponding values of correlation coefficients in males were 0.449 (p< 0.001), -0.030 (NS), 0.221 (p = 0.008), and 0.432 (p< 0.001), respectively.

**Figure 9:**
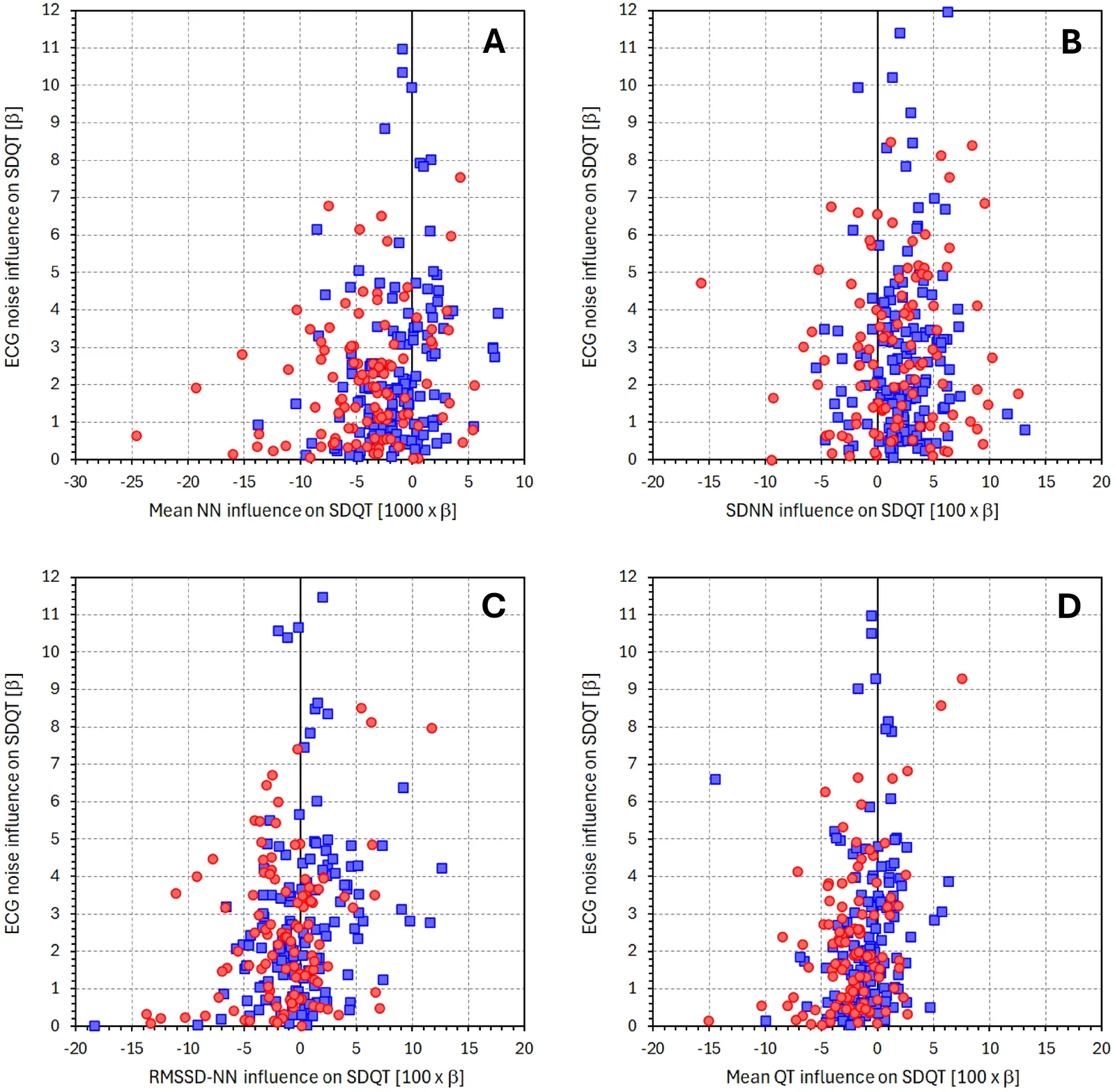
Results of the bivariable regression analyses relating SDQT values to the combination of ECG noise with mean NN intervals **(A)**, SDNN **(B)**, RMSSD-NN **(C)**, and mean QT intervals **(D)**. The layout of all panels and meaning of symbols correspond to the layout and symbols of Panel B of **Figure 1**. In all panels, the regression contributions of ECG noise are shown on the vertical axis, the contributions of the other covariate on the horizontal axis.

Cumulative distributions of the bivariate SDQT *β* coefficients are shown in **Figure 10**. Note that while in the univariable analysis, there were cases where the noise-related *β* coefficient was negative, in the bivariable analyses, all these *β* coefficients of ECG noise were positive. The Figure also shows that the distributions of the noise-related *β* coefficients showed only very tiny differences between females and males. The *β* coefficients of the other SDQT covariates were statistically significantly different (p< 0.001, p = 0.003, and p< 0.001 for mean NN, RMSSD-NN, and mean QT, respectively) except of the SDNN-related coefficients that showed somewhat peculiar distributions.

**Figure 10:**
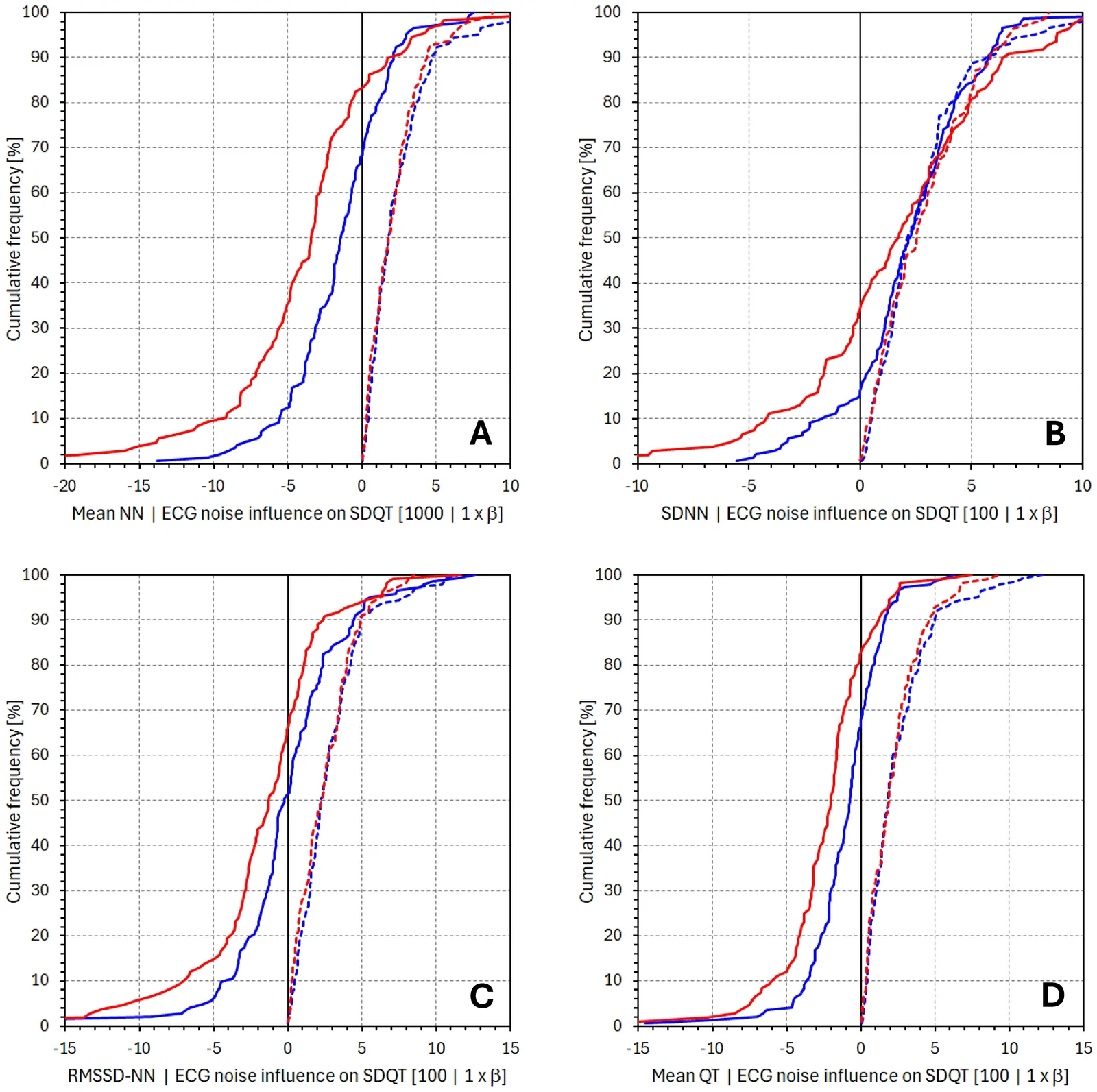
Results of the bivariable regression analyses relating SDQT values to the combination of ECG noise with mean NN intervals **(A)**, SDNN **(B)**, RMSSD-NN **(C)**, and mean QT intervals **(D)**. The layout of all panels and meaning of symbols correspond to the layout and symbols of Panel D of **Figure 1**. In all panels, the dashed lines correspond to the contributions of ECG noise, the full lines to the contributions of the other covariate.

**Figure 11** shows the results of the final step of the bivariable SDQT regressions after the backwards eliminations were applied. In females (central pie-charts of individual panels of **Figure 11**) the counts of subjects with significant covariates were as follows. Panel A: mean NN 45 (41.7%), ECG noise 53 (49.1%), overlap 20 (18.5%); Panel B: SDNN 32 (29.6%), ECG noise 62 (57.4%), overlap 25 (23.1%); Panel C: RMSSD-NN 27 (25%), ECG noise 59 (54.6%), overlap 16 (14.8%); Panel D: mean QT 45 (41.7%), ECG noise 53 (49.1%), overlap 20 (18.5%). In males (surrounding donut-charts in **Figure 11**) the corresponding counts were as follows. Panel A: mean NN 54 (37.8%), ECG noise 72 (50.3%), overlap 18 (12.6%); Panel B: SDNN 62 (43.4%), ECG noise 80 (55.9%), overlap 39 (27.3%); Panel C: RMSSD-NN 36 (25.2%), ECG noise 88 (61.5%), overlap 24 (16.8%); Panel D: mean QT 49 (34.3%), ECG noise 74 (51.7%), overlap 17 (11.9%).

**Figure 11:**
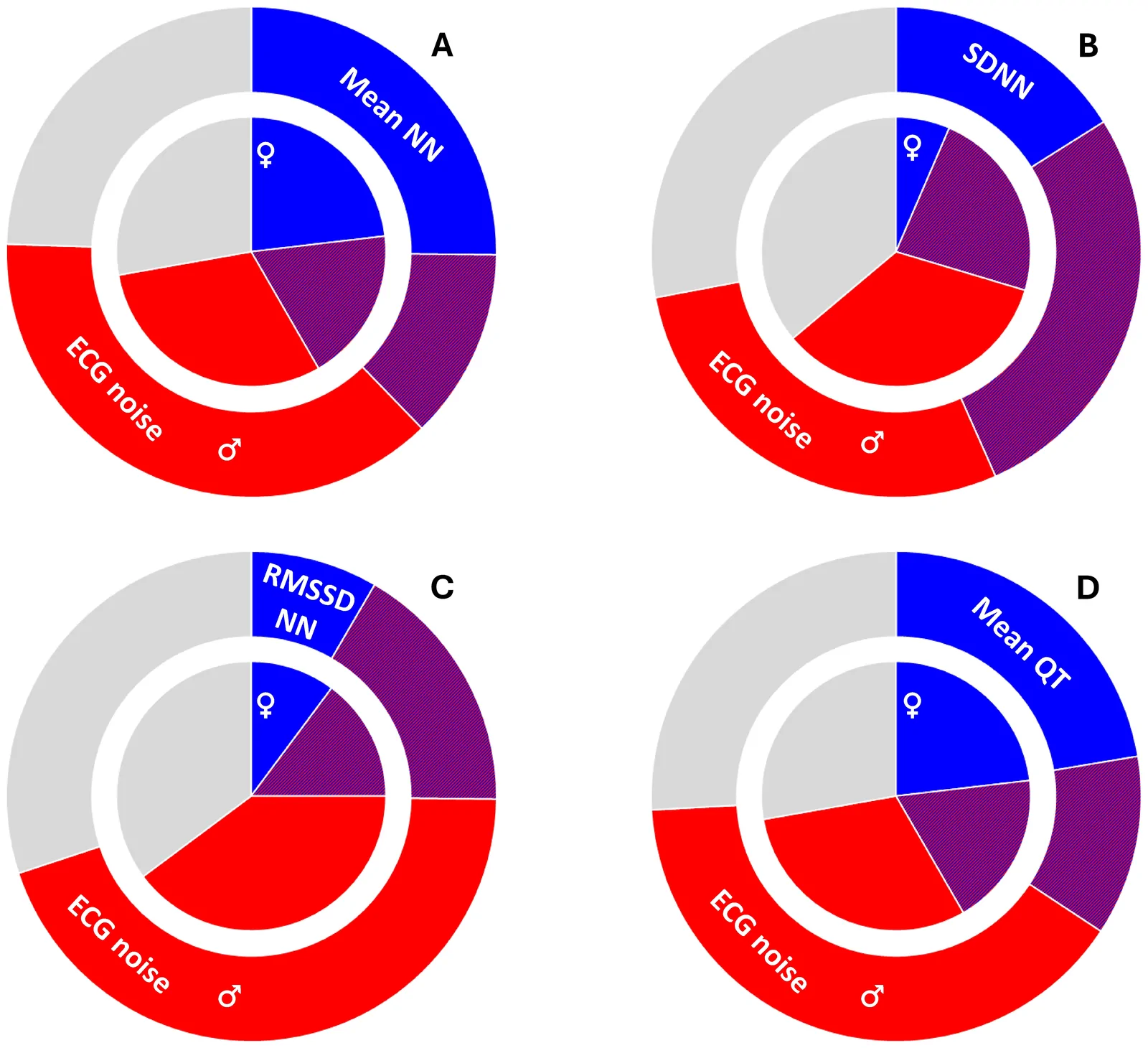
Results of the bivariable regression analyses relating SDQT values to the combination of ECG noise with mean NN intervals **(A)**, SDNN **(B)**, RMSSD-NN **(C)**, and mean QT intervals **(D)**. Each panel is composed of a central pie-chart that corresponds to female subjects and of a surrounding donut-chart that corresponds to male subjects. Both the central pie chart and the surrounding donut chart indicate the proportion of subject in whom only the ECG noise was statistically significant in the regression analysis (red sector of the charts), subjects in whom only the other covariate was significant (blue sectors), subjects in whom both the ECG noise and the other covariate were significant (violet sectors), and subjects in whom SDNN was significantly related to neither ECG noise nor the other covariate (light grey sectors).

These numbers confirm the observation that in both sexes and in all 4 bivariate regressions, the numbers of subjects in whom ECG noise contributed significantly to SDQT were larger than the number of subjects with a significant contribution by other covariates.

### Bivariable regressions of RMSSD-QT

The results of bivariate analysis of RMSSD-QT are presented in **Supplementary Figures 1** to **S5** which present the same steps of the analysis as **Figures 7** to **11**. The observations were very similar to those of the bivariate analysis of SDQT. Again, as seen in the **Supplementary Figure 5**, in both sexes and in all 4 bivariate regressions, the numbers of subjects in whom ECG noise contributed significantly to RMSSD-QT were larger than the number of subjects with a significant contribution by other covariates.

### Trivariable regressions

The final results of the trivariable regression analyses of SDQT covariates are shown graphically in **Figure 12** (results for females and males shown in the inner and outer donut-charts of individual panels, respectively). Importantly, even when using triplets of covariates and excluding non-significant covariates during the backwards elimination, ECG noise remained statistically significant in appreciable proportions of subjects. These proportions were as follows. Panel A: females 42 (35.6%), males 51 (38.3%); Panel B: females 53 (44.9%), males 65 (48.9%); Panel C: females 57 (48.3%), males 69 (51.9%); Panel D: females 48 (40.7%), males 50 (37.6%).

**Figure 12:**
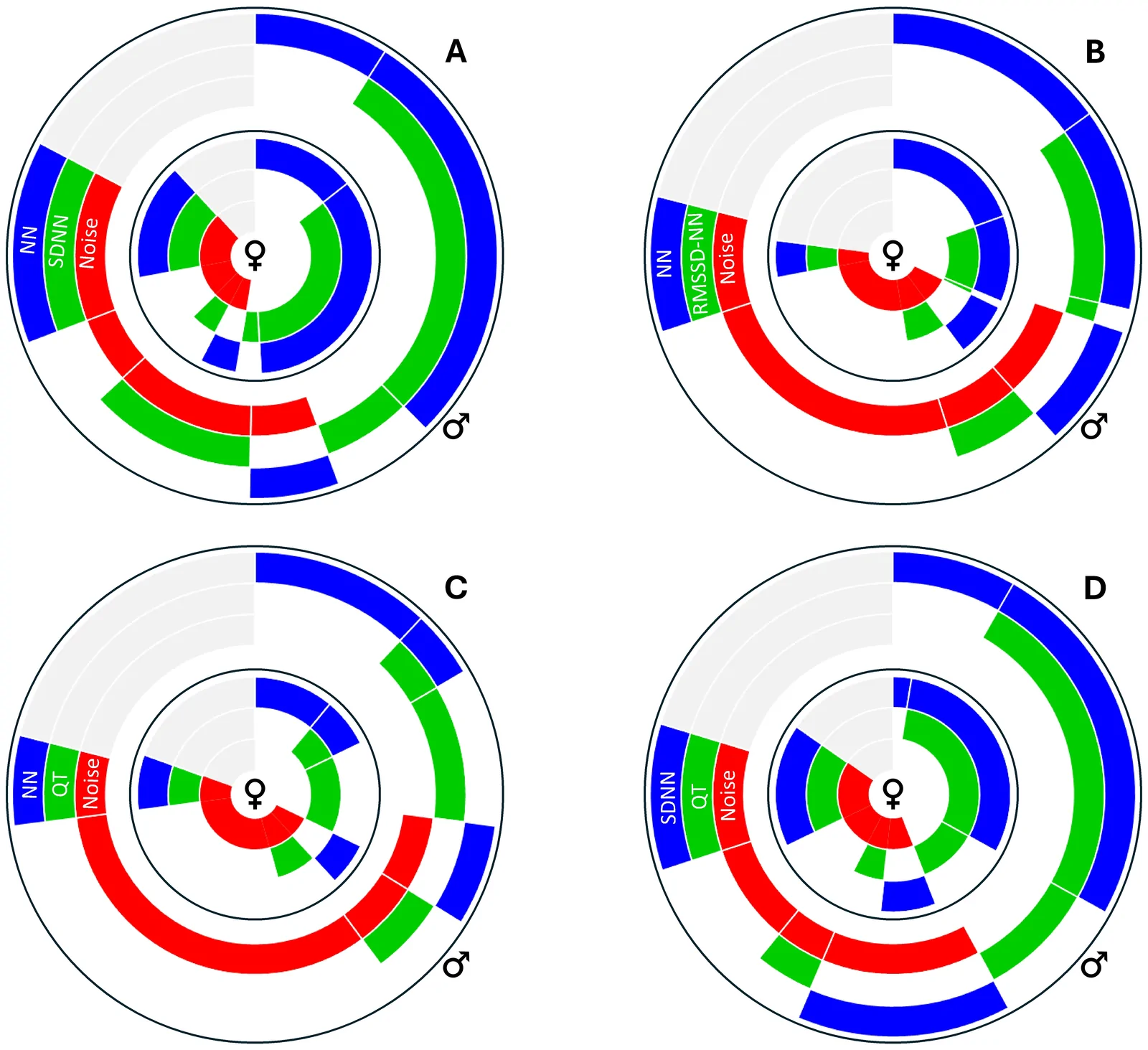
Results of trivariable regression analyses that related SDQT to a combination of mean NN + SDNN + ECG noise **(A)**, mean NN + RMSSD-NN + ECG noise **(B)**, mean NN + mean QT + ECG noise **(C)** and SDNN + mean QT + ECG noise **(D)**. Each panel is composed of an inner and outer stack of donut-charts that correspond to female and male subjects, respectively. Each stack is composed of three donut-charts that correspond to the individual covariates involved in the regression analysis (see the labels at the left side of the outer stack). Each donut-chart corresponds to the total population of the given sex and indicates subjects for whom the covariate remained significant in the trivariable regression analysis. The light grey sectors of the donut charts correspond to subjects in whom SDQT was significantly related to none of the investigated covariates.

As seen in **Figure 13**, very similar results were also obtained with trivariable regression analyses of RMSSD-QT. The numbers of subjects for whom the ECG noise remained statistically significant after backwards covariate elimination were as follows. Panel A: females 55 (46.6%), males 57 (42.9%); Panel B: females 62 (52.5%), males 62 (46.6%); Panel C: females 64 (54.2%), males 64 (48.1%); Panel D: females 66 (55.9%), males 64 (48.1%).

**Figure 13:**
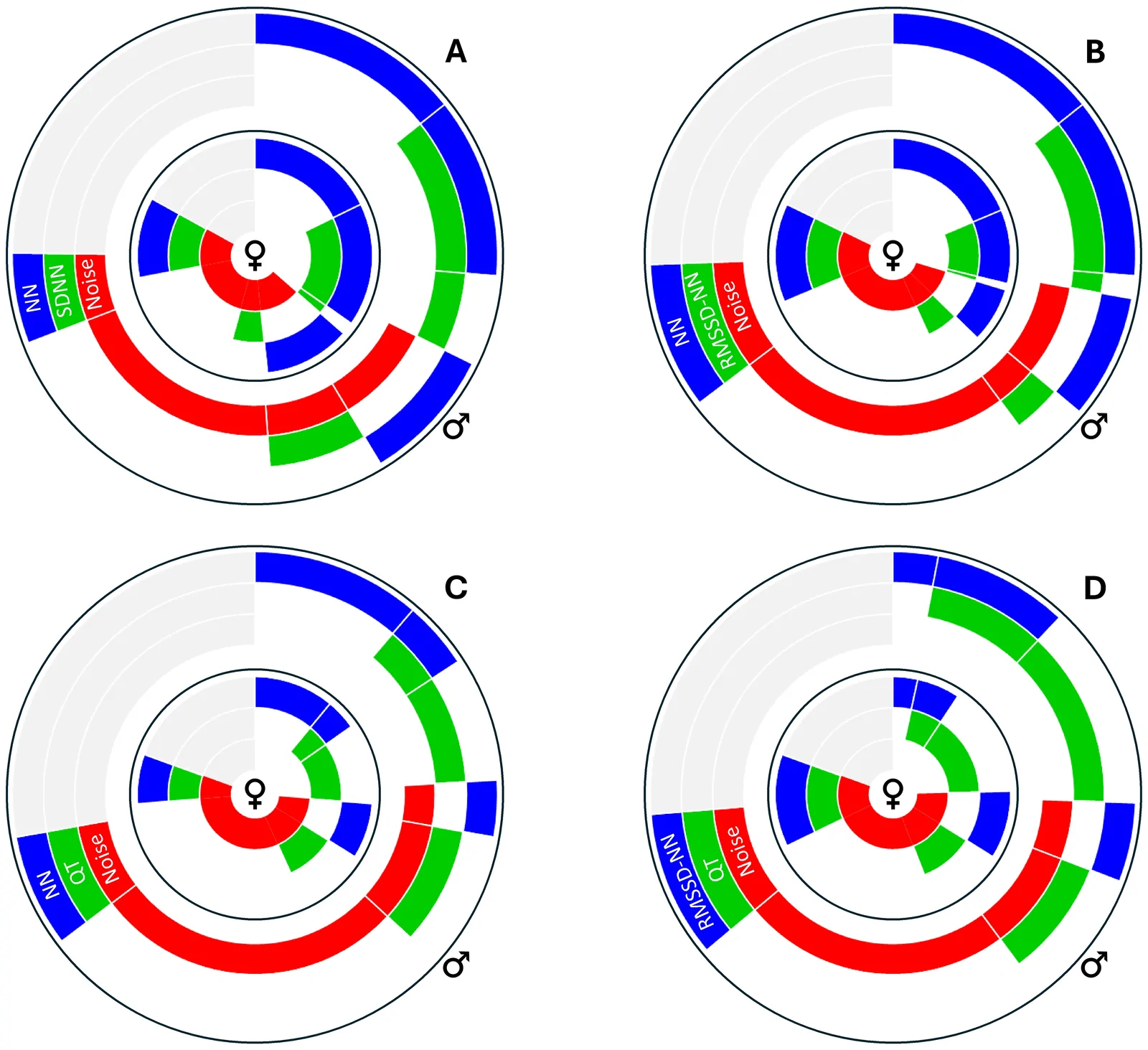
Results of trivariable regression analyses that related RMSSD-QT to a combination of mean NN + SDNN + ECG noise **(A)**, mean NN + RMSSD-NN + ECG noise **(B)**, mean NN + mean QT + ECG noise **(C)** and RMSSD-NN + mean QT + ECG noise **(D)**. The layout and meaning of the panels are the same as the meaning layout and meaning of panels in **Figure 12**.

Comparisons of **Figures 12** and **13** suggests that while the influence of ECG noise was substantial for both SDQR and RMSSD-QT, it was more expressed for RMSSD-QT.

### Full multivariable analyses

The results of full multivariable analyses are shown in **Figure 14** (SDQT regression analyses) and in **Figure 15** (RMSSD-QT regression analyses).

**Figure 14:**
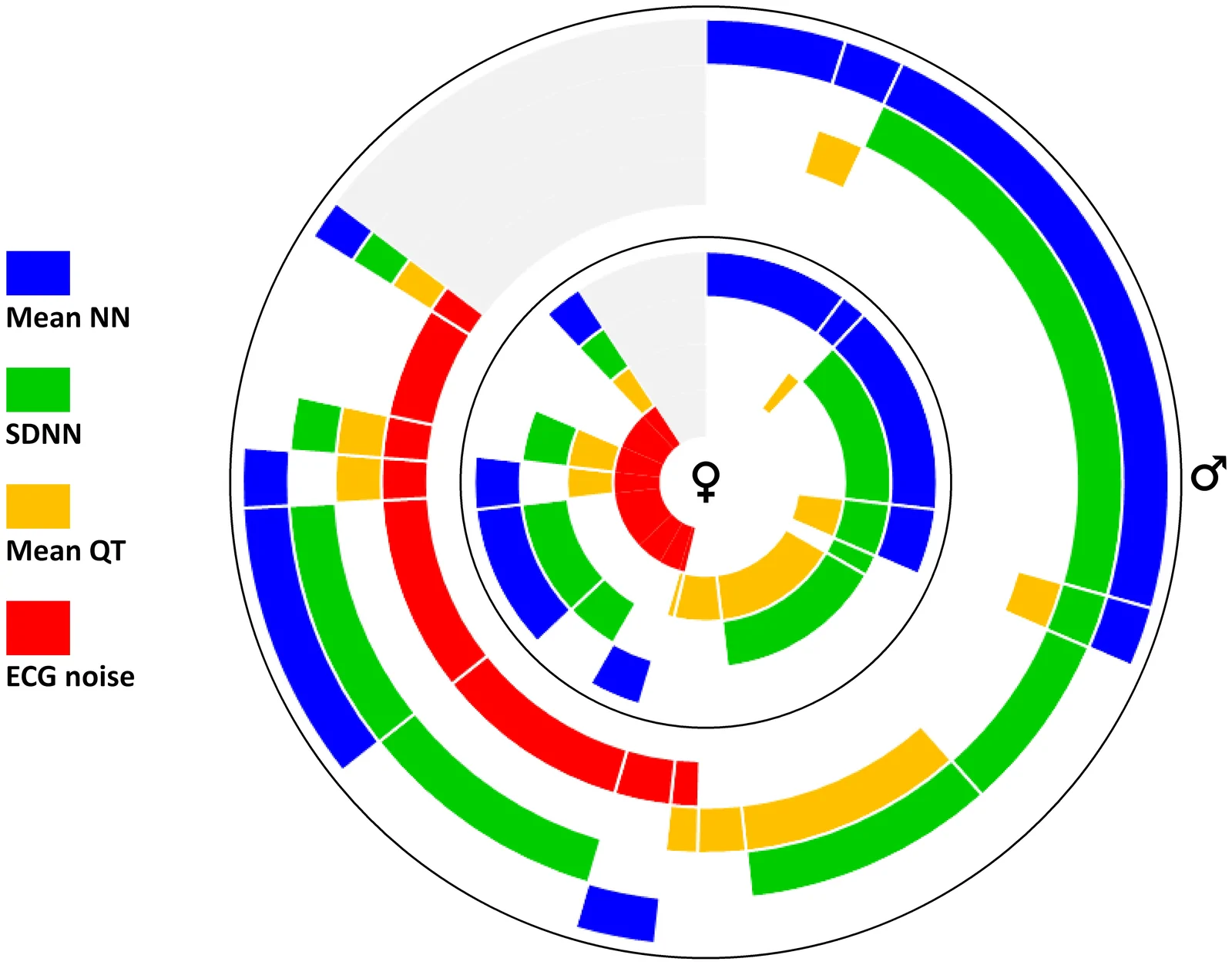
Results of full multivariable regression analysis that related SDNQT to a combination of 4 covariates, that is mean NN + SDNN + mean QT + ECG noise. The figure is composed of inner and outer stack of donut-charts that correspond to female and male subjects, respectively. Each of the stacks consists of 4 donut-charts that represent the investigated covariates (see the legend). Each donut-chart corresponds to the total population of the given sex (i.e., each radial line across the donut-chart stack represents one subject) and indicates subjects for whom the given covariate remained significant in the full multivariable regression analysis. The light grey sectors of the donut charts correspond to subjects in whom SDQT was significantly related to none of the covariates.

The counts of females for whom the individual covariates survived the multivariable SDQT analysis were: mean NN 56 (51.9%), SDNN 63 (58.3%), mean QT 42 (38.9%), ECG noise 40 (37.0%), no significant covariates 10 (9.3%). For males, these counts were: mean NN 68 (47.6%), SDNN 92 (64.3%), mean QT 33 (23.1%), ECG noise 50 (35.0%), no significant covariates 21 (14.7%). **Figure 14** shows the corresponding overlaps.

The results for multivariable RMSSD-QT analyses were similar. Counts for females: mean NN 44 (40.7%), RMSSD-NN 35 (32.4%), mean QT 37 (34.3%), ECG noise 54 (50.0%), no significant covariates 15 (13.9%). Counts for males: mean NN 44 (30.8%), RMSSD-NN 42 (29.4%), mean QT 45 (31.5%), ECG noise 69 (48.3%), no significant covariates 32 (22.4%). Again, **Figure 15** shows the corresponding overlaps.

**Figure 15:**
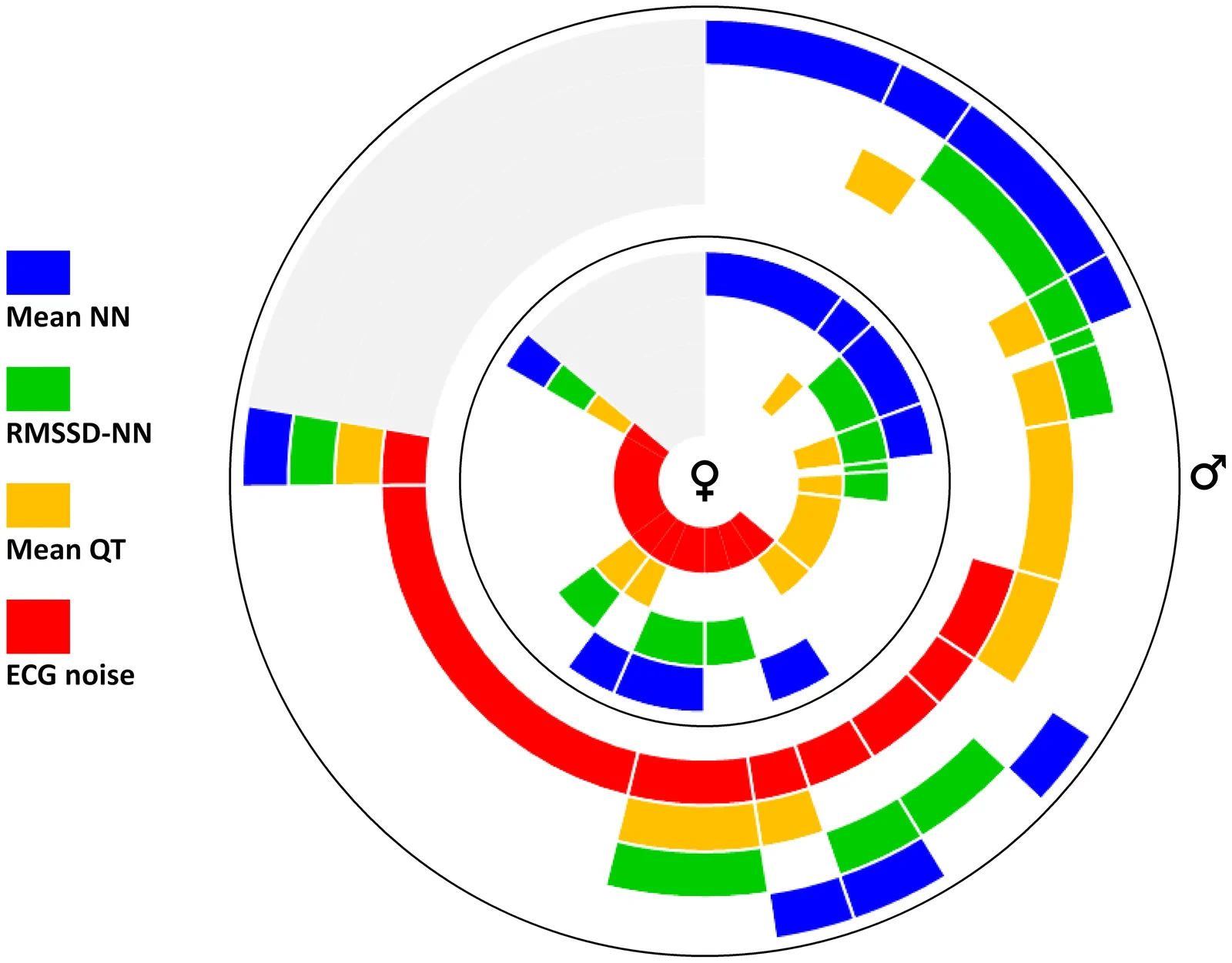
Results of full multivariable regression analysis that related RMSSD-QT to a combination of 4 covariates, that is mean NN + RMSSD-NN + mean QT + ECG noise. The figure is composed of inner and outer stack of donut-charts that correspond to female and male subjects, respectively. Each of the stacks consists of 4 donut-charts that represent the investigated covariates (see the legend). Each donut-chart corresponds to the total population of the given sex (i.e., each radial line across the donut-chart stack represents one subject) and indicates subjects for whom the given covariate remained significant in the full multivariable regression analysis. The light grey sectors of the donut charts correspond to subjects in whom SDQT was significantly related to none of the covariates.

Thus, even when relating QT variability expressions to a combination of mean NN interval, relevant HRV indices, and mean QT interval, the influence of ECG noise was far from eliminated.

## Discussion

The presented results allow classifying the observations into several areas.

The ECG noise data well below 10% prove that the data selection for the present investigation was very successful as far as the elimination of data with poor signal to noise ratios. At the same time, the comparison of the results shown in **Figures 1** and **2** shows that the ECG noise data make physiological sense in terms of the relationship to heart rate and SDNN.

Despite the elimination of cases with substantial ECG noise pollution, the performed analyses document that ECG noise was a substantial and frequently dominant covariate for both SDQT and RMSSD-QT. This observation is consistent with previous reports (Avbelj et al., 2003; Eskola et al., 2005; Toman et al., 2022) and confirms the well-known difficulty with accurate delineation of the T wave end as documented by a multitude of methods devoted to the problem (Eskola et al., 2005; Chen et al., 2006; Noriega et al., 2012; Rahul and Sharma, 2021). Attention should therefore be given to the methods for the assessment of T wave morphology which is less likely dependent on the minute details of T wave delineation. Nevertheless, although numerous methods for the assessment of T wave morphology have been proposed (Acar et al., 1999; Hnatkova et al., 2001; Ono et al., 2006; Porthan et al., 2009; Isaksen et al., 2021), no systematic investigation on their dependence on ECG noise has been undertaken or at least reported.

Even in these data that were selected to secure high signal-to-noise ratios, the effects of noise on basic QT variability measurements were erratic when comparing the results of individual subject-specific analyses. This is not surprising since even minor ECG signal imprecisions might easily lead to overestimation as well as underestimation of the QT interval duration even when using the cross-correlation and other techniques (Berger et al., 1997; Hnatkova et al., 2009) aimed at eliminating the effects of localised signal disturbances. The ECG noise thus likely results from a combination of both physiological and technical mechanisms. Their separation is problematic based on ECG signal alone. Nevertheless, the increases of QT variability with increasing heart rate (**Figures 5**, **6**) allow making the following suggestions. On the one hand, known pro-arrhythmic effects of increased sympathetic tone might be responsible for repolarisation instability that manifests also on a beat-to-beat basis. On the other hand, activations of skeletal muscles that trigger homeostatic demands for increased heart rate contribute to myopotential pollution of ECG signals. This leads, independently of the measurement technology employed, to lower accuracy of the ECG interval measurements. In future studies, independent myopotential recordings on body surface might assist the accuracy estimates of ECG interval durations.

These observations further stress the importance of developing different techniques and matrices for the characterisation of ECG repolarisation signals. Although most previous attempts concentrated on T wave morphology in individual leads, ECG analysis techniques that use advanced algebraic methods and consider all ECG leads of the standard recordings together in multidimensional spaces have been shown promising in the analyses involving QRS complexes (Hnatkova et al., 2022; Hnatkova et al., 2024; Řehoř et al., 2026b). It seems logical to expect that such analytical methods would prove similarly effective in the assessment of repolarisation signals and their beat-to-beat variability. Indeed, for some of the analytical methods, this has already been shown (Rahola et al., 2024).

The results, in some respects, contradict the formula for QT variability index (Berger et al., 1997), i.e. the method for correcting SDQT for a combination of heart rate, SDNN, and QT duration. As seen in **Figure 14**, the combination of mean NN, SDNN, and mean QT was only rarely contributing to SDQT with statistical significance of all 3 covariates (only in 7.4% of females and in 3.5% of males, irrespective of whether the ECG noise did or did not also reach statistical significance). Among other aspects, the QT variability index formula divides SDQT by QT mean (Řehoř et al., 2026a). This concept is not supported by our observations since we have shown (see panels C of **Figures 5**, **6**) that in most cases, mean QT interval relates to SDQT negatively. Similarly, the irregular nature of the SDNN influence on SDQT (**Figure 5B**) makes it questionable whether SDQT might simply be divided by SDNN (Řehoř et al., 2026a). Since reduced SDNN is a known powerful predictor of adverse outcome (Task Force of the European Society of Cardiology and the North American Society of Pacing and Electrophysiology, 1996; Steger et al., 2021; Jarczok et al., 2022) it is possible that the reported predictive value of increased QT variability index was, to a large degree, merely reflecting the predictive value of HRV. Indeed, when we tested the regression composite of QT variability index [defined by the published formula (Berger et al., 1997; Baumert et al., 2016)] in the data of this investigation (details not shown), we observed that in the vast majority of subjects, QT variability index was significantly related to mean NN and/or SDNN. QT variability index does not therefore serve as a correction of SDQT for heart rate and HRV as it does not provide data independent of heart rate and HRV.

The influence of ECG noise also explains why we are finding it impractical to correct standard QT variability indices for heart rate (or mean of NN intervals) and/or basic HRV parameters. When SDQT and RMSSD-QT are not only strongly but also inconsistently (in terms of inter-subject differences) dependent on ECG signal quality, it is not surprising to observe a substantial variability of intra-subject regression coefficients as we have shown in **Figure 5B**. This variability of the regression *β* coefficients means that any fixed correction formula, i.e., a variant of QT variability index formula calculating corrected values (SDQT)c, might be effective in some subjects but would be very ineffective in others. Similar problem is well-known in the correction of QT interval duration for underlying heart rate and leads to inaccuracies in fixed QTc corrections (Hnatkova and Malik, 1999; Malik et al., 2019; Andršová et al., 2021) and to the application of individual QTc correction techniques (Sciot et al., 2016; Kleiman et al., 2026).

This analogy might lead to considerations of proposing subject-specific corrections of QT variability indices [perhaps using similarly advanced technologies as those proposed for accurate individual-specific QTc corrections (Malik et al., 2013)]. Nevertheless, such an approach would also require reasonably stable intra-subject relationship of QT variability to recording ECG noise which not only makes no physiologic and/or technical sense but is also contradicted by our observations of substantial inter-subject variability of regression *β* coefficients expressing the ECG noise influence (**Figures 5D**, **9**). Indeed, ECG noise represents recording components that, despite likely having some biological component, cannot be expected to characterise a sufficiently reproducible physiologic quantity. That is, the ECG noise levels are not directly reproducible components of QT variability. Increased noise levels merely contribute to the inaccuracies in the interpretation of the duration of ECG waves. The higher the noise, the less accurate and thus less reproducible estimates of QT interval duration. While it is possible to measure the noise content objectively, it is not possible to express objectively individual errors made in the QT interval measurement of any given ECG beat. Indeed, if errors of individual measurements were measurable, they could be eliminated before the “true” variability is expressed numerically. In other words, there is not only noise in the ECG but also a different and unquantifiable “noise” in the ECG interpretation. We can only postulate that the technically measured noise of ECG recordings correlates with the interpretation inaccuracy. This is insufficient for any correction purposes because despite the correlation, it is impossible to distinguish between the factual component of QT variability and the additional error-driven component due to signal noise pollution.

The same considerations apply to the proportional evaluation of our results. The signal-to-noise ratio that we used to express the ECG noise numerically is a surrogate of the artificial part of QT variability. Since the proportion of the valid and artificial parts is not known (and, in principle, cannot be known) in data based solely on ECG measurements, it is not surprising that the relationship to technically expressed noise is highly variable between different subjects.

Practical implications of our results are complicated by anecdotal observations that reduced ECG signal quality appears to be a clinical risk predictor in its own right, which is possibly consistent with the skin effects of sympathetic tone (Everett et al., 2017; Borle et al., 2024; Yu et al., 2024). This means that as ECG noise influences QT variability, the predictive value of QT variability might not directly indicate the involvement of repolarisation abnormalities. In future studies of QT variability, ECG noise needs to be considered. Inclusion of noise levels in multivariable regression analyses would help strengthening study outcomes. By this we mean that increased ECG noise levels (i.e., lowered signal to noise ratio) might represent approximate estimates of inaccuracies imposed on the ECG measurement. If, in comparison to QT variability expressions, ECG noise levels lead to similar (or even stronger) statistical differences in study outcomes, the validity of the observations might require additional scrutiny.

Naturally, we are not dismissing the published observations on the predictive value of QT variability indices (Baumert et al., 2016) but believe that more careful physiological interpretations of these results are warranted and that the measurement techniques should be scrutinised in detail. The lessons from the concept of QT dispersion should not be forgotten (Rautaharju, 1999; Malik and Batchvarov, 2000).

### Limitations

The use of data and ECG recordings of healthy subjects restricts the implications of our study for clinical populations. Nevertheless, it allowed us to concentrate on physiologic and technical aspects that are likely present in other ECG studies. If ECG noise influences the precision of QT variability measurements in healthy subjects, it is difficult to suppose that the same impact would be absent in clinical ECG collections. Intentionally, we have used separate regression models in individual study subjects rather than mixed effect regression modelling. Mixed effect regression modelling would assume that the influence of individual covariates (that is, also of ECG noise) follows the same physiologic principle in different subjects. This was impossible to assume. While we suggest that the variability of T wave morphology might effectively replace the assessment of QT interval variability, we have no data to show that T wave morphology analyses are not similarly influenced by ECG recording quality. As described, we have intentionally excluded subjects with lower quality recordings and missing data. This might have biased the results presented in this text. It is unlikely that the exclusion of ECGs with low signal-to-noise levels contributed to our observations but the true relationship between QT variability indices and ECG noise might have been underestimated. The use of linear regression models assumes the relationship between a variable and its covariates to be reasonably monotonic. It was outside the scope of this study to investigate the validity of these assumptions. Nevertheless, it seems logical to expect that the inaccuracy of ECG measurements increases steadily with ECG noise levels. The number of datapoints available for each subject was sufficient for the applied regression analyses but insufficient to design tests of more advanced relationships since the type of statistical distribution of ECG noise was impossible to be estimated beforehand. Using more general regression models would likely provide results inconsistent between individual subjects.

## Conclusion

The results of the study permit to conclude that it is not feasible to reduce the inconsistency of expressions of QT variability by correcting them for other electrocardiographic measurements such as heart rate or HRV parameters. Simple QT variability indices such as SDQT or RMSSD-QT are highly influenced by ECG signal quality. When QT variability measures are used in studies of risk prediction, influence of ECG noise needs to be carefully considered. Thus, the signal-to-noise ratios of the source ECG recordings should also be investigated and their values used in multivariable risk regressions as indices competing with QT interval variability.

## Data Availability

The original contributions presented in the study are included in the article/**Supplementary Material**. Further inquiries can be directed to the corresponding author.
