# Supplementary material for: Intra-subject covariates of QT variability indices: influence of ECG noise
Source: Front Physiol. 2026 Sep 21;17:1926685. doi: 10.3389/fphys.2026.1926685 (PMC13634824; doi:10.3389/fphys.2026.1926685)
Supplement: Supplementary file 1 [file DataSheet1.pdf]

Intra-subject covariates of QT variability indices:

Influence of ECG noise

by

Jan Řehoř, Kateřina Helánová, Martina Šišáková, Tomáš Novotný,

Irena Andršová, Marek Malik

Supplementary Material

Supplementary Table 1

Parameter	Position	Females	Males	p-value
Mean NN [ms]	Supine	868.8 ± 88.2	932.2 ± 90.5	7.05×10^{-8}
	Sitting	753.7 ± 82.2	800.3 ± 81.2	1.21×10^{-5}
	Standing	615.0 ± 83.4	650.0 ± 81.2	9.99×10^{-4}
SDNN [ms]	Supine	44.15 ± 13.55	47.45 ± 13.87	6.03×10^{-2}
	Sitting	39.65 ± 10.94	44.42 ± 12.49	1.48×10^{-3}
	Standing	29.42 ± 9.27	34.18 ± 10.04	1.31×10^{-4}
RMSSD-NN [ms]	Supine	38.47±16.54	37.77±17.12	7.45×10^{-1}
	Sitting	26.56±9.84	27.15±12.54	6.80×10^{-1}
	Standing	12.93±5.67	15.53±7.50	1.98×10^{-3}
Mean QT [ms]	Supine	392.5 ± 18.3	385.2 ± 17.1	1.43×10^{-3}
	Sitting	372.6 ± 20.2	364.6 ± 16.1	8.63×10^{-4}
	Standing	348.9 ± 23.9	345.0 ± 18.1	1.65×10^{-1}
SDQT [ms]	Supine	2.975 ± 0.687	2.788 ± 0.646	3.00×10^{-2}
	Sitting	3.720 ± 1.126	3.299 ± 0.832	1.27×10^{-3}
	Standing	4.682 ± 1.592	3.695 ± 0.895	3.78×10^{-8}
RMSSD-QT [ms]	Supine	2.424±0.716	2.215±0.696	2.13×10^{-2}
	Sitting	3.228±1.204	2.757±1.019	1.24×10^{-3}
	Standing	3.745±1.484	2.978±1.058	9.01×10^{-6}

Population averages of measured ECG data in different postural positions. p-value column shows the significances of the sex comparisons.

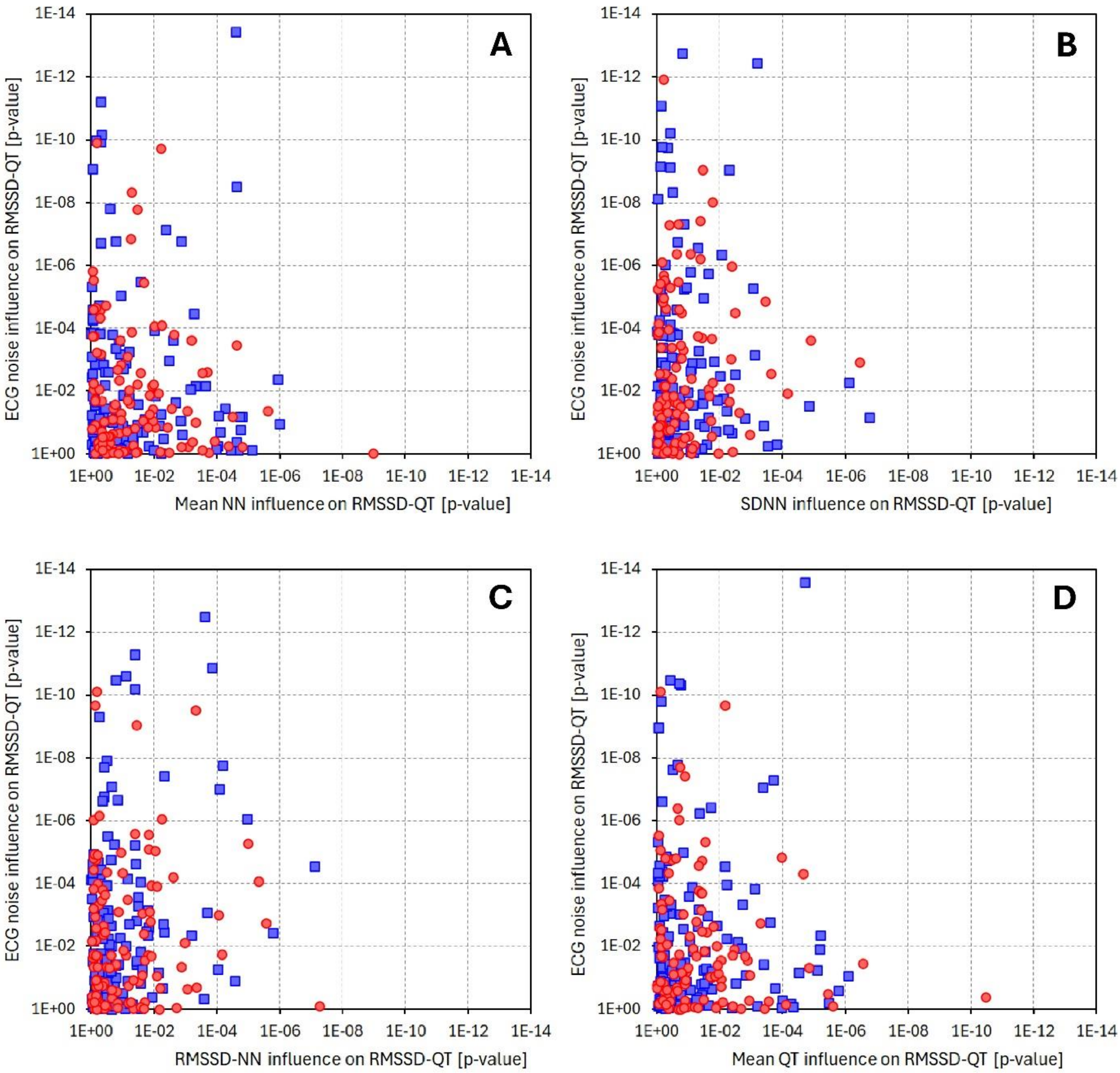

Figure S1

Results of the bivariable regression analyses relating RMSSD-QT values to the combination of ECG noise with mean NN intervals (Panel A), SDNN (panel B), RMSSD-NN (Panel C), and mean QT intervals (Panel D). The layout of all panels and meaning of symbols correspond to the layout and symbols of Panel A of Figure 1. In all panels, the regression contributions of ECG noise are shown on the vertical axis, the contributions of the other covariate on the horizontal axis.

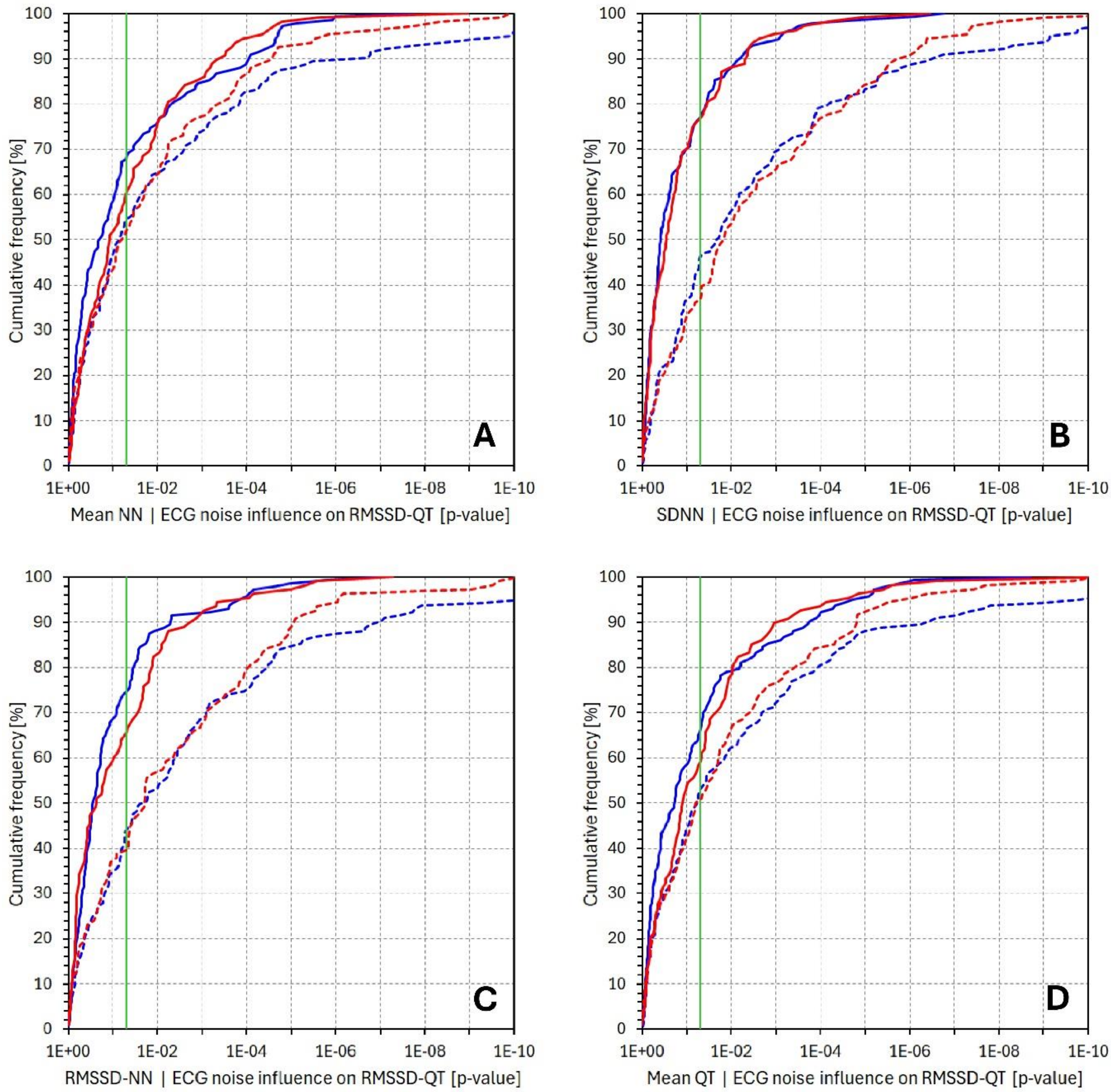

Figure S2

Results of the bivariable regression analyses relating RMSSD-QT values to the combination of ECG noise with mean NN intervals (Panel A), SDNN (panel B), RMSSD-NN (Panel C), and mean QT intervals (Panel D). The layout of all panels and meaning of symbols correspond to the layout and symbols of Panel C of Figure 1. In all panels, the dashed lines correspond to the contributions of ECG noise, the full lines to the contributions of the other covariate.

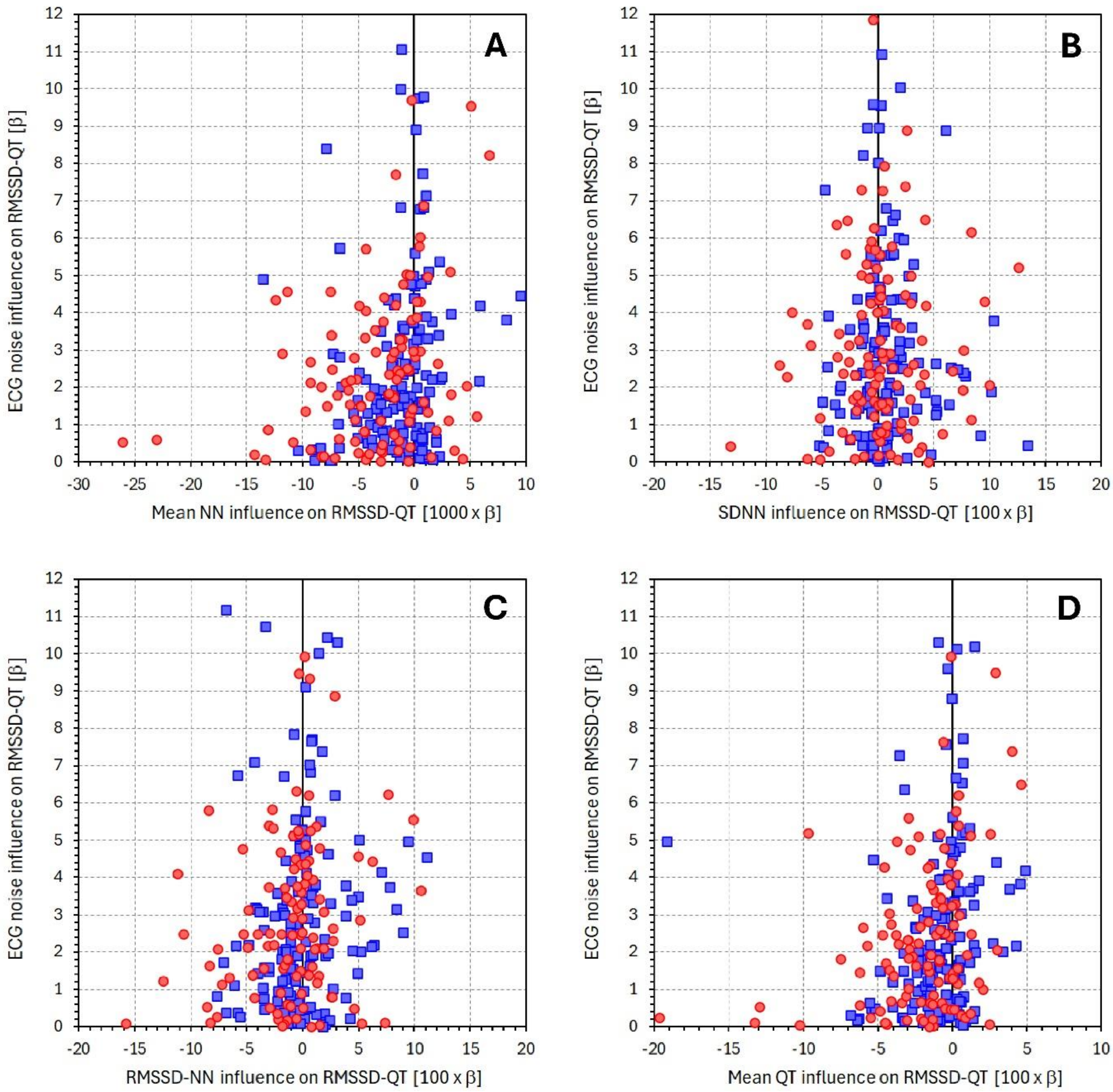

Figure S3

Results of the bivariable regression analyses relating RMSSD-QT values to the combination of ECG noise with mean NN intervals (Panel A), SDNN (panel B), RMSSD-NN (Panel C), and mean QT intervals (Panel D). The layout of all panels and meaning of symbols correspond to the layout and symbols of Panel B of Figure 1. In all panels, the regression contributions of ECG noise are shown on the vertical axis, the contributions of the other covariate on the horizontal axis.

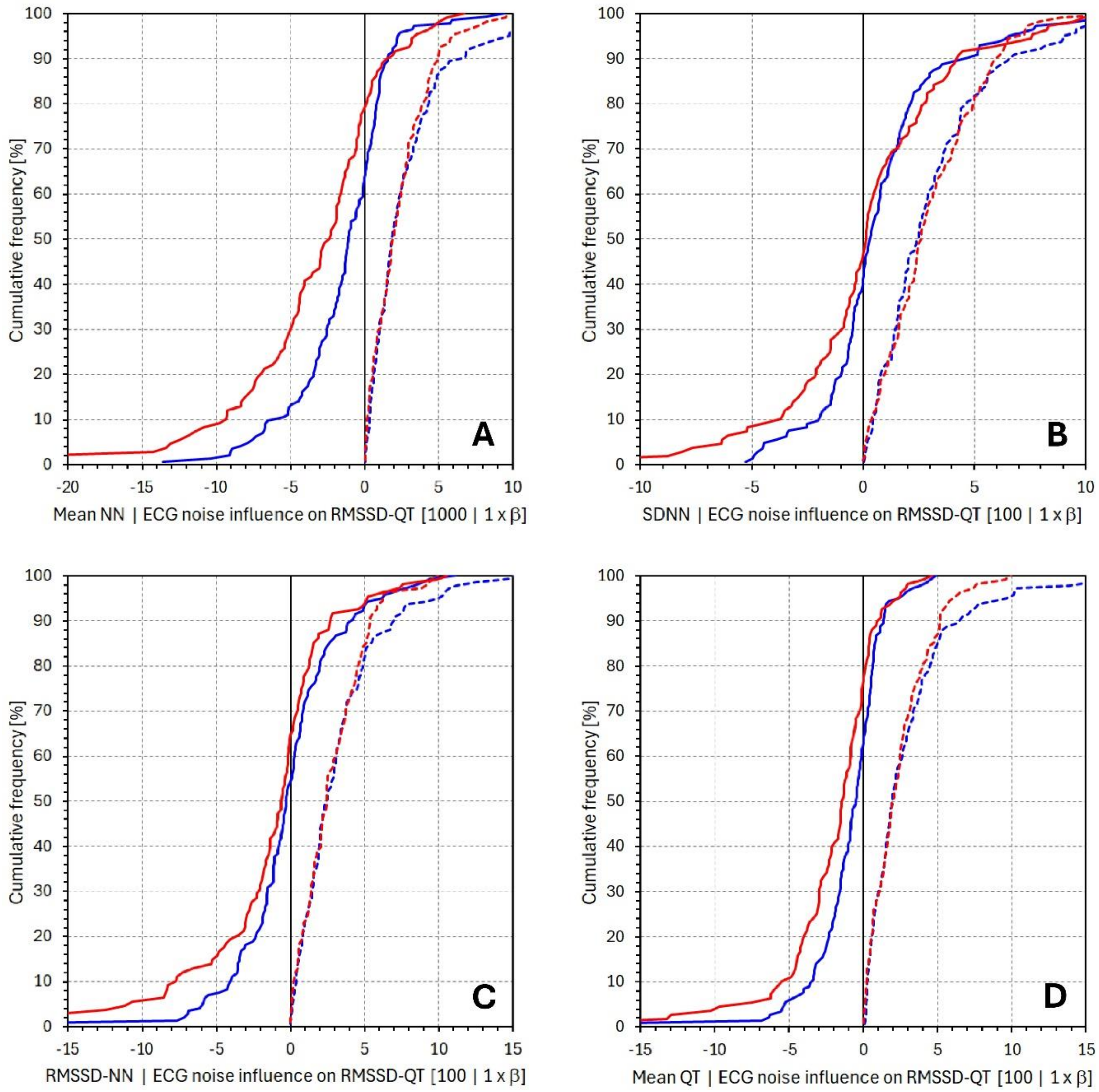

Figure S4

Results of the bivariable regression analyses relating RMSSD-QT values to the combination of ECG noise with mean NN intervals (Panel A), SDNN (panel B), RMSSD-NN (Panel C), and mean QT intervals (Panel D). The layout of all panels and meaning of symbols correspond to the layout and symbols of Panel D of Figure 1. In all panels, the dashed lines correspond to the contributions of ECG noise, the full lines to the contributions of the other covariate.

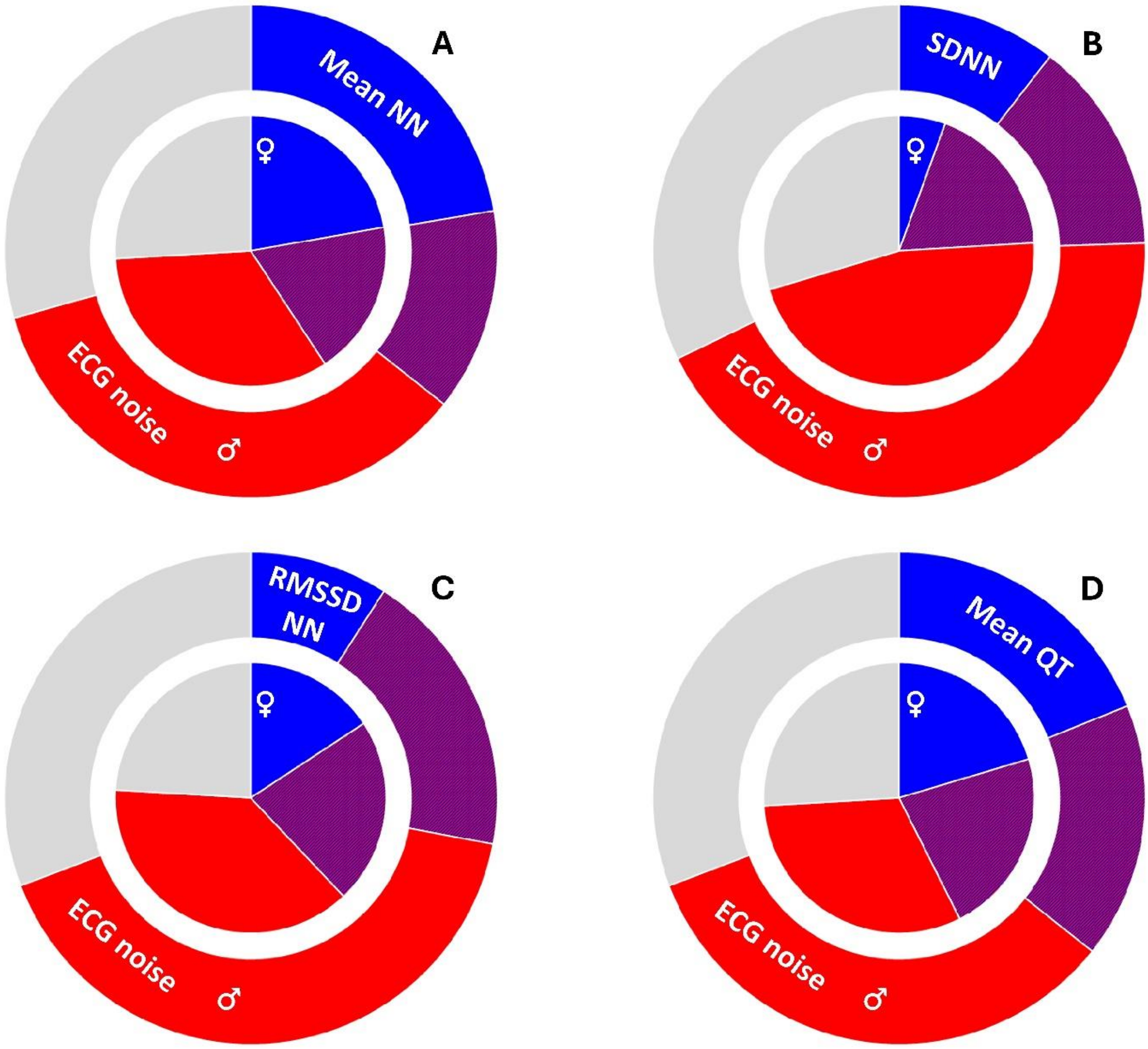

Figure S5

Results of the bivariable regression analyses relating RMSSD-QT values to the combination of ECG noise with mean NN intervals (Panel A), SDNN (panel B), RMSSD-NN (Panel C), and mean QT intervals (Panel D). Each panel is composed of a central pie-chart that corresponds to female subjects and of a surrounding donut-chart that corresponds to male subjects. Both the central pie chart and the surrounding donut chart indicate the proportion of subjects in whom only the ECG noise was statistically significant in the regression analysis (red sector of the charts), subjects in whom only the other covariate was significant (blue sectors), subjects in whom both the ECG noise and the other covariate were significant (violet sectors), and subjects in whom RMSSD-QT was significantly related to neither ECG noise nor the other covariate (light grey sectors).
